# Live-cell tracking of γ-H2AX kinetics reveals the distinct modes of ATM and DNA-PK in the immediate response to DNA damage

**DOI:** 10.1242/jcs.260698

**Published:** 2023-04-27

**Authors:** Watanya Trakarnphornsombat, Hiroshi Kimura

**Affiliations:** ^1^ Department of Life Science and Technology, School of Life Science and Technology, Tokyo Institute of Technology, Yokohama 226-8501, Japan; ^2^ Cell Biology Center, Institute of Innovative Research, Tokyo Institute of Technology, Yokohama 226-8503, Japan

**Keywords:** ATM, DNA-PK, DNA repair, H2AX, DNA double-strand breaks

## Abstract

DNA double-strand breaks (DSBs) are a serious form of DNA damage that can cause genetic mutation. On the induction of DSBs, histone H2AX becomes phosphorylated by kinases, including ataxia telangiectasia-mutated (ATM), ataxia telangiectasia and Rad3-related (ATR) and DNA-dependent protein kinase (DNA-PK). Phosphorylated H2AX (γ-H2AX) can be a platform to recruit DNA repair machinery. Here, we analyzed the immediate early kinetics of γ-H2AX upon laser-induced DNA damage in ATM-proficient and -deficient living cells by using fluorescently labeled antigen-binding fragments specific for γ-H2AX. The accumulation kinetics of γ-H2AX were similar in both ATM-proficient and -deficient cells. However, γ-H2AX accumulation was delayed when the cells were treated with a DNA-PK inhibitor, suggesting that DNA-PK rapidly phosphorylates H2AX at DSB sites. Ku80 (also known as XRCC5), a DNA-PK subunit, diffuses freely in the nucleus without DNA damage, whereas ATM repeatedly binds to and dissociates from chromatin. The accumulation of ATM at damage sites was regulated by the histone H4K16 acetyltransferase MOF (also known as KAT8 in mammals), but its accumulation was not necessarily reflected in the γ-H2AX level. These results suggest distinct actions of ATM and DNA-PK in immediate γ-H2AX accumulation.

## Teaser

**Summary:** Live-cell imaging reveals that DNA-dependent protein kinase, but not ATM, plays a primary role in the phosphorylation of histone H2AX during the immediate early response to DNA damages.

## INTRODUCTION

DNA double-strand breaks (DSBs) are one of the most deleterious forms of DNA damage because even a single DSB can activate the DNA damage checkpoint, which delays cell cycle progression (van den Berg et al., 2018) and triggers cell death (Rich et al., 2000). These DSBs are occur naturally, with only 10–50 events per cell per day (Gospodinov and Ugrinova, 2019; Tubbs and Nussenzweig, 2017; Vilenchik and Knudson, 2003), but they threaten genomic integrity, which is essential for regulation of cellular homeostasis and the maintenance of genetic information. If the DSB repair process is not properly performed, various types of mutations can arise, which might eventually lead to diseases, such as cancer (Negrini et al., 2010; Tubbs and Nussenzweig, 2017) and aging (Tian et al., 2019).

In the DSB repair response, protein kinases belonging to the phosphatidylinositol 3-kinase-related kinase (PIKK) family, including ataxia telangiectasia-mutated (ATM), ataxia telangiectasia and Rad3-related (ATR), and DNA-dependent protein kinase (DNA-PK), have critical roles. These kinases phosphorylate various proteins involved in DSB repair and the histone H2A variant H2AX at serine 139 (Blackford and Jackson, 2017). Serine 139-phosphorylated H2AX, called γ-H2AX (Rogakou et al., 1998), facilitates the concentration of DNA damage repair machinery (Celeste et al., 2003) and serves as a DNA damage signal (Hunt et al., 2013). Large-scale proteomics analysis has identified >700 proteins that are phosphorylated by ATM and ATR upon ionizing radiation, demonstrating that multiple protein networks are involved in DNA damage repair and signaling processes (Matsuoka et al., 2007). The PIKK family kinases have both redundant and distinct functions. ATM and DNA-PK function in response to DSBs throughout the cell cycle, and ATR functions mostly in DNA replication-associated damage during S phase (Gospodinov and Ugrinova, 2019; Riabinska et al., 2013). Even though these kinases all prefer to phosphorylate a serine or threonine residue that is followed by a glutamine (SQ/TQ motif) (Blackford and Jackson, 2017), their knockout phenotypes are different. ATM-knockout mice are sterile and often suffer from lymphopenia, whereas DNA-PK-knockout mice are fertile and have a severe combined immunodeficiency (SCID) phenotype (Menolfi and Zha, 2020). It has been proposed that ATM promotes γ-H2AX clustering and DNA repair accuracy, whereas DNA-PK is essential for end joining (Caron et al., 2015). ATM is also known to phosphorylate threonine 392 of the protein males absent on the first (MOF; also known as KAT8 in mammals) (Gupta et al., 2014), which is a histone acetyltransferase for H4 lysine 16 acetylation (H4K16ac) (Sharma et al., 2010; Taipale et al., 2005) that assists in chromatin decompaction and facilitates recruitment of DNA repair machinery, including homologous recombination (HR) repair proteins (Dhar et al., 2017; Gupta et al., 2014; Horikoshi et al., 2019; Hunt et al., 2013; Kim et al., 2019; Sharma et al., 2010). However, it remains unclear how ATM and DNA-PK function in γ-H2AX formation just after DSBs are generated.

The dynamics of γ-H2AX have been analyzed by immunofluorescence, immunoblotting and chromatin immunoprecipitation (Burma et al., 2001; Caron et al., 2015; Stiff et al., 2004), but the γ-H2AX formation kinetics that occur immediately after DSBs (within a few minutes) in single cells have not been elucidated because of the lack of a monitoring system for γ-H2AX in living cells. However, by introducing fluorescently labeled modification-specific antigen-binding fragments (Fabs) into living cells, changes in the target modifications can be tracked (Conic et al., 2018; Hayashi-Takanaka et al., 2011; Sato et al., 2021). In the present study, we used γ-H2AX-specific Fabs (Yamagata et al., 2019) to analyze the kinetics of γ-H2AX formation in response to laser-induced DNA damage in living human cells. We first compared the involvement of ATM in immediate γ-H2AX kinetics. After laser microirradiation, γ-H2AX accumulated at the damaged areas with similar kinetics in ATM-proficient and -deficient cells. In contrast, the inhibition of DNA-PK activity slowed the γ-H2AX accumulation and dissolution kinetics. Fluorescence recovery after photobleaching (FRAP) and permeabilized cell assays revealed that the DNA-PK subunit Ku80 (also known as XRCC5) diffuses freely throughout the nucleus, whereas ATM repeatedly binds to and unbinds from chromatin. Thus, DNA-PK can bind DNA ends immediately after DSB formation and phosphorylate H2AX, whereas ATM appears to be dispensable for immediate early γ-H2AX accumulation in DSBs.

## RESULTS

### Kinetics of γ-H2AX immediately after DSB induction

**Fig. 1.**
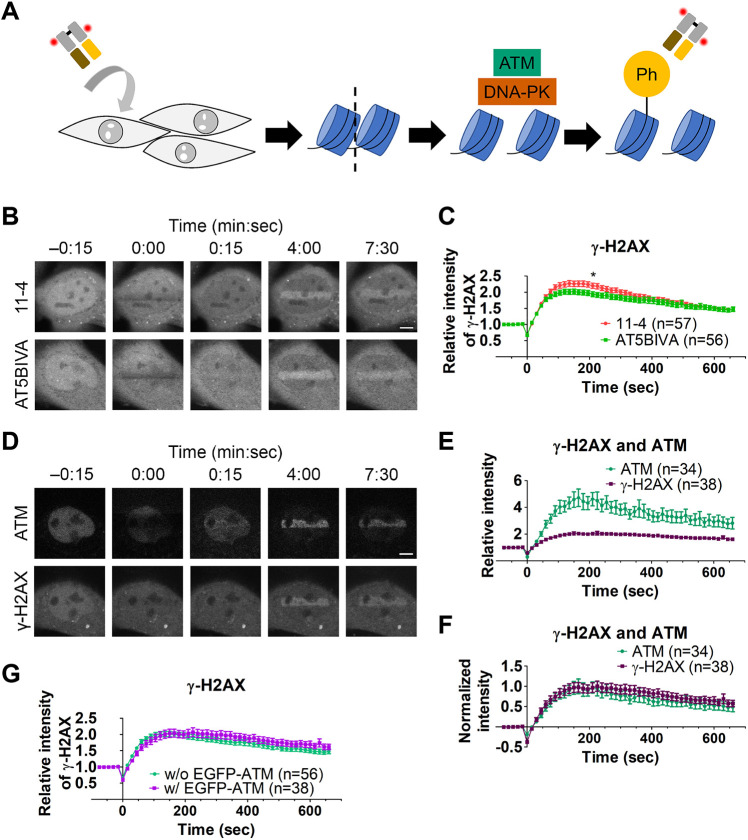
**Dynamics of ATM and γ-H2AX after DSB induction by 405-nm laser bleaching.** (A) Schematic diagram of the experimental system showing how the formation of phosphorylated H2AX (γ-H2AX) in response to a DSB is detected in living cells. A fluorescent dye-conjugated γ-H2AX-specific Fab is loaded into cells, and then DNA damage is induced by 405-nm laser irradiation. ATM and DNA-PK are recruited to DNA lesions and phosphorylate histone H2AX to form γ-H2AX, which is now recognized by the Fab. Thus, the localization and degree of γ-H2AX can be monitored by live-cell imaging. (B,C) Monitoring of γ-H2AX in response to laser microirradiation in ATM-proficient (11-4) and -deficient (AT5BIVA) cells. Cy5-conjugated γ-H2AX Fab had been loaded into 11-4 and AT5BIVA cells and rectangular areas were then irradiated to induce DSBs. (B) Time-lapse images. In the irradiated areas, fluorescence was bleached just after irradiation (0:00) and then increased (4:00 and 7:30). (C) Accumulation kinetics of γ-H2AX. The relative intensities of γ-H2AX Fab in the irradiated areas in 11-4 and AT5BIVA cells are plotted (mean±s.e.m., with the total number of cells indicated as *n* from three replicates; see Fig. S1B,C for data in individual replicates). *P<0.05 (*P*=0.056 at 105 s, *P*=0.019 at 210 s, and *P*=0.066 at 315 s; unpaired, two-tailed Student's *t*-test). (D–F) Monitoring EGFP–ATM and γ-H2AX in response to laser microirradiation. (D) Time-lapse images. AT5BIVA cells that express EGFP–ATM plasmid were loaded with Cy5-conjugated γ-H2AX Fab and rectangular areas were irradiated to induce DSBs. Both EGFP–ATM and γ-H2AX accumulated in the irradiated areas. (E,F) The accumulation kinetics of EGFP–ATM and γ-H2AX in the irradiated area. (E) The relative intensities (mean±s.e.m., with the total number of cells indicated as *n* from two replicates). (F) Normalized intensities from the baseline before irradiation to the maximum (*P*=0.906 at 105 s, *P*=1.000 at 210 s, and *P*=0.351 at 315 s; unpaired, two-tailed Student's *t*-test). (G) Comparison of the accumulation kinetics of Cy5-conjugated γ-H2AX Fab in irradiated areas in AT5BIVA cells without (w/o) and with (w/) EGFP–ATM, reproduced from data in C and E. No significant difference is seen (*P*=0.477 at 105 s, *P*=0.485 at 210 s, and *P*=0.183 at 315 s; unpaired, two-tailed Student's *t*-test). Scale bars: 5 μm.

To examine the immediate early kinetics of γ-H2AX in response to DNA damage, we loaded fluorescently labeled γ-H2AX-specific Fabs, which have previously been used to detect DSBs in mouse embryos (Yamagata et al., 2019), into cells and microirradiated a part of the nucleus using a 405-nm laser to induce DSBs (Muster et al., 2017) (Fig. 1A). We first compared the kinetics in ATM-deficient (AT5BIVA) and -proficient (11-4) cells (both of which are of human origin). AT5BIVA cells harbor an in-frame deletion in the kinase domain of the ATM gene (Cheema et al., 2013; Gilad et al., 1996), resulting in little ATM protein expression (Fig. S1A). The 11-4 cells were derived from AT5BIVA by transferring a chromosome 11 that expresses the functional ATM (Komatsu et al., 1990) (Fig. S1A). Before laser microirradiation, Cy5-labeled γ-H2AX Fabs were distributed throughout the cytoplasm and nucleus (except for the nucleoli) in both AT5BIVA and 11-4 cells (Fig. 1B, −0:15 min:sec). Just after 405-nm laser irradiation, the fluorescence in the irradiated area was decreased by photobleaching (0:00) and then increased over the nuclear background within minutes (0:15, 4:00 and 7:30). The relative fluorescence intensities of γ-H2AX Fab in the irradiated area were measured and plotted from three independent experiments (Fig. 1C; Fig. S1B,C). In both ATM-deficient (AT5BIVA) and -proficient (11-4) cells, γ-H2AX Fabs accumulated in the irradiated area, reaching a broad peak at ∼100–200 s and then gradually declining (Fig. 1C). The γ-H2AX Fabs showed slightly higher accumulation in the 11-4 cells than in the AT5BIVA cells.

To compare the accumulation kinetics of ATM and γ-H2AX in damaged areas, EGFP-tagged ATM (EGFP–ATM) was expressed in AT5BIVA cells. Accumulation in the irradiated area was greater for EGFP–ATM than for γ-H2AX (Fig. 1D,E), but when normalized by setting the maximum intensity at 1 and the original intensity at 0, the accumulation kinetics of EGFP–ATM and γ-H2AX were similar (Fig. 1F). The kinetics of γ-H2AX accumulation did not change in the AT5BIVA cells without or with EGFP–ATM expression (Fig. 1G). These data suggest that ATM is not essential for H2AX phosphorylation immediately after DSBs were induced by laser irradiation, although the accumulation of ATM coincided with the γ-H2AX kinetics.

We next determined whether the kinetics of γ-H2AX accumulation differ in different cell cycle phases, because ATR is known to function during the S phase. In addition, the accumulation kinetics could be affected by the preference as to which DSB repair pathway is used, either HR or non-homologous end joining (NHEJ), which depends on the cell cycle (Her and Bunting, 2018; Karanam et al., 2012; Shrivastav et al., 2008). For a cell cycle marker in living cells, we used PCNA tagged with a fluorescent protein, which shows characteristic distributions depending on the cell cycle phase (Essers et al., 2005; Leonhardt et al., 2000; Mir et al., 2011; Schönenberger et al., 2015; Somanathan et al., 2001). mCherry–PCNA was diffuse in both the cytoplasm and nucleus during most of the G1 phase, concentrated on foci during the S phase, and diffuse in the nucleus during the G2 phase (Fig. S2A,B) (Uchino et al., 2022). The accumulation kinetics of γ-H2AX Fabs were essentially similar in all cell cycle phases (Fig. S2C–G), with slightly more accumulation, albeit insignificantly, in 11-4 cells than AT5BIVA cells, as observed without mCherry-PCNA expression (Fig. 1C).

### Effects of the specific inhibitors of ATM, ATR and DNA-PK on H2AX phosphorylation

The finding that similar γ-H2AX accumulation kinetics were observed with and without ATM suggests that other PIKK family kinases (ATR and/or DNA-PK) might have a primary role in immediate H2AX phosphorylation or compensate for ATM function in deficient cells. To determine which of these PIKK family kinases is a primary kinase for immediate γ-H2AX formation, we first determined the effective concentration of kinase-selective inhibitors in AT5BIVA and 11-4 cells by immunofluorescence. The cells were incubated with an ATR inhibitor (AZ20), an ATM inhibitor (KU55933) or a DNA-PK inhibitor (NU7441) at 10, 5, 2.5, and 0 μM, simultaneously with 20 μg/ml etoposide (ETP) to induce DSBs, for 1 h, before fixing and staining with γ-H2AX-specific antibody (Fig. S3A–C). In ETP-treated 11-4 cells, γ-H2AX signals were observed at similar levels in the presence of a single inhibitor (Fig. S3D). In AT5BIVA cells, γ-H2AX fluorescence intensity was drastically decreased with the DNA-PK inhibitor, but not with the ATR and ATM inhibitors (Fig. S3E). Susceptibility to the DNA-PK inhibitor in AT5BIVA cells was rescued by EGFP–ATM expression (Fig. S3F). This result is consistent with a previous study showing that in the absence of functional ATM, γ-H2AX formation after DSBs was primarily mediated through DNA-PK (Stiff et al., 2004). When DNA-PK activity is inhibited, ATM could phosphorylate H2AX in 60 min (Fig. S3).

**Fig. 2.**
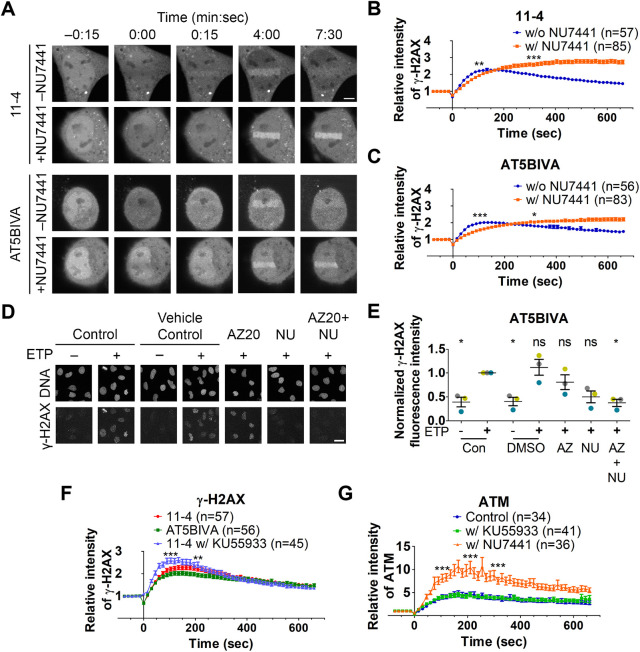
**Effect of ATM and DNA-PK inhibitor on γ-H2AX dynamics.** (A–C) Loading of 11-4 and AT5BIVA cells with Cy5-conjugated γ-H2AX Fab; cells were then treated with or without 2.5 μM NU7441 for ≥1 h before and during the laser-irradiation assay. (A) Time-lapse images. Scale bar: 5 μm. (B,C) Accumulation kinetics of γ-H2AX Fab at the irradiated area (mean±s.e.m., with the total number of cells indicated as *n* from three and five replicates). (B) 11-4 cells (*P*=0.009 at 105 s, *P*=0.060 at 210 s, and *P*<0.0001 at 315 s; unpaired, two-tailed Student's *t*-test). (C) AT5BIVA cells (*P*<0.0001 at 105 s, *P*=0.661 at 210 s, and *P*=0.039 at 315 s; unpaired, two-tailed Student's *t*-test). (D,E) AT5BIVA cells were treated with 1 μM AZ20 and/or 2.5 μM NU7441 for 1 h, then 20 μg/ml ETP was added for 20 min, before staining with γ-H2AX-specific antibody. (D) Fluorescence images. Scale bar: 25 μm. (E) Relative fluorescence intensity (mean±s.e.m.; *n*=3 independent experiments indicated in different colors; ≥101 cells were analyzed in each experiment and the fluorescence intensity was normalized). Statistical significance compared to the control cells treated with ETP (Con+) is indicated (one-way ANOVA test with Tukey test as the post hoc analysis). (F) 11-4 cells were treated with 5 μM KU55933 for ≥1 h before and during the laser-irradiation assay (mean±s.e.m., with the number of cells indicated as *n* from two or three replicates). Graphs of untreated controls are reproduction of Fig. 1C for comparison and statistical analysis was performed with non-treated 11-4 data (*P*<0.0001 at 105 s, *P*=0.003 at 210 s, and *P*=0.201 at 315 s; one-way ANOVA test with Tukey test as the post hoc analysis). (G) AT5BIVA cells expressing EGFP–ATM were treated with 5 μM KU55933 or 2.5 μM NU7441 for ≥1 h before and during the laser-irradiation assay (mean±s.e.m., with the number of cells indicated as *n* from two or three replicates). The graph of untreated control is a reproduction of Fig. 1E for comparison (KU55933 versus control; *P*=1.000 at 105 s, *P*=0.998 at 210 s, and *P*=1.000 at 315 s; NU7441 versus control, *P*<0.0001 at 105, 210 and 315 s; one-way ANOVA test with Tukey test as the post hoc analysis). w/, with; w/o, without. *P<0.05; **P<0.01; ***P<0.001; ns, not significant.

To analyze the effect of DNA-PK on immediate early γ-H2AX formation kinetics, cells were incubated with 2.5 μM NU7441 for ≥1 h before and during the laser-irradiation assay. To minimize the contribution of ATR, we chose G1 cells for the analysis using cells stably expressing mCherry–PCNA. In 11-4 cells treated with NU7441, γ-H2AX Fab continually accumulated for ∼500 s, in contrast to the decrease after ∼200 s in control cells without the inhibitor (Fig. 2A,B). The delayed decrease might have been caused by ATM hyperactivation, which can be induced by DNA-PK inhibitors (Zhou et al., 2017) or by impaired DNA repair. In AT5BIVA cells treated with NU7441, in which both ATM and DNA-PK activities are diminished, γ-H2AX Fab accumulation was substantially reduced compared to that in control ATM-deficient cells (Fig. 2A,C). This result indicates that DNA-PK activity is critical in the early response to DSBs. When AT5BIVA cells were treated with AZ20 and NU7441 to inhibit both ATR and DNA-PK, the γ-H2AX level was reduced to the same level as in control cells without DNA damage (Fig. 2D,E), which suggests that the low level of γ-H2AX accumulation in NU7441-treated AT5BIVA cells (Fig. 2A,C) is mediated by ATR (Bøe et al., 2018).

To confirm that the early γ-H2AX dynamics were similar regardless of the presence of ATM, 11-4 cells were treated with the ATM inhibitor, KU55933, before and during laser irradiation. Unexpectedly, γ-H2AX accumulated more rapidly in KU55933-treated 11-4 cells compared to that seen in untreated 11-4 and AT5BIVA cells (Fig. 2F), whereas EGFP–ATM accumulated similarly without and with KU55933 (Fig. 2G, blue and green curves). This indicates that treatment with the ATM-specific inhibitor does not phenocopy the ATM protein-deficient cells, as previously suggested (Choi et al., 2010). Although the mechanism of how the chemical inhibition of ATM stimulates γ-H2AX formation in the early response remains unknown, DNA-PK accumulates slightly more with KU55933 (see below). In the presence of the DNA-PK inhibitor NU7441, EGFP–ATM accumulated much more (Fig. 2G), suggesting that the inhibition of one of ATM and DNA-PK can facilitate the accumulation of each other through a compensation mechanism. However, in the presence of the DNA-PK inhibitor, a substantial time-lag was observed for γ-H2AX accumulation kinetics despite more ATM being accumulated. From this observation, it is likely that the accumulation and activation of ATM are not coupled.

### Involvement of MOF in the early DNA damage response

Crosstalk between γ-H2AX and another histone modification, H4K16ac, has been previously demonstrated. H4K16ac is mediated through the action of the histone acetyltransferase MOF (Sharma et al., 2010; Taipale et al., 2005) and is involved in DSB repair (Dhar et al., 2017; Horikoshi et al., 2019; Kim et al., 2019; Miller et al., 2010). H4K16ac obstructs the binding of 53BP1 (also known as TP53BP1) to H4K20me2 (Tang et al., 2013) and regulates the DNA repair pathway choice by limiting DNA end resection, which is a required step for HR (Pellegrino et al., 2017). ATM phosphorylates MOF to facilitate HR protein recruitment (Gupta et al., 2014), and MOF promotes ATM kinase activity (Gupta et al., 2005; Li et al., 2010). Therefore, we analyzed the possible involvement of H4K16ac in early γ-H2AX kinetics. We first used the specific Fab to determine if H4K16ac is accumulated in laser-irradiated areas. After the induction of DSBs, H4K16ac Fab did not show obvious accumulation in either AT5BIVA or 11-4 cells regardless of the presence of the DNA-PK inhibitor, NU7441 (Fig. S4). This suggests that H4K16ac levels do not change several min after DSB induction.

**Fig. 3.**
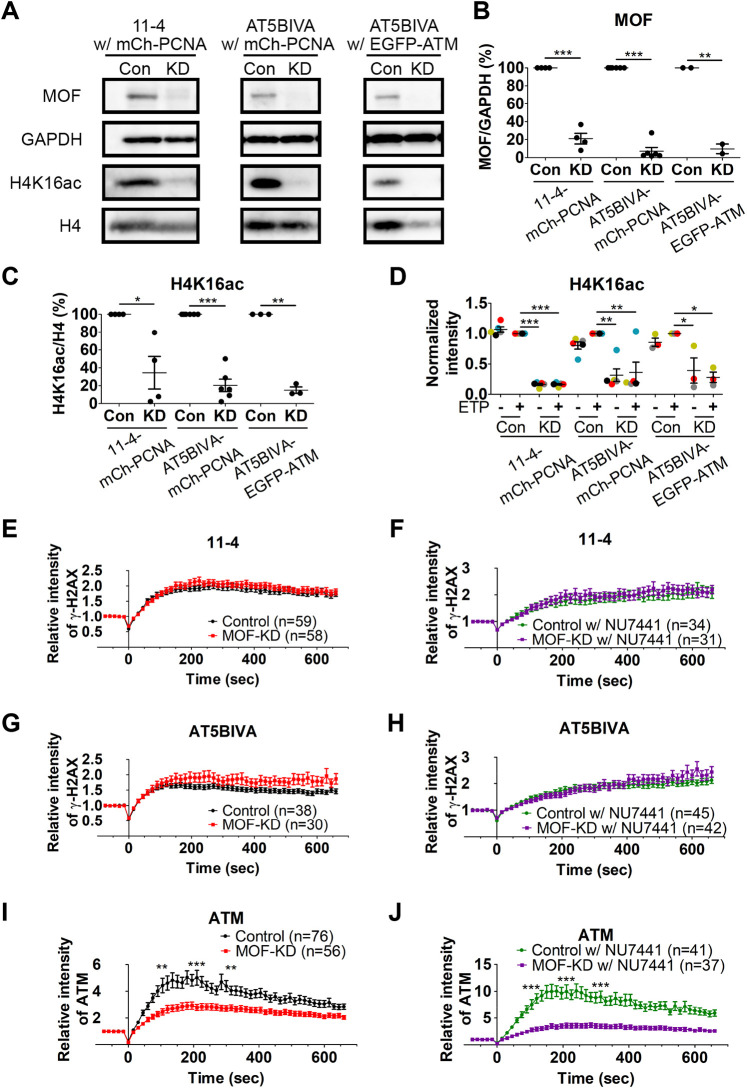
**Effects of MOF knockdown on the accumulation of γ-H2AX.** Lentivirus to cause expression of MOF-specific (KD) and control-scramble shRNA (Con) was used to infect 11-4 and AT5BIVA cells that expressed mCherry (mCh)–PCNA, or AT5BIVA cells that also expressed EGFP–ATM. (A–D) Validation of MOF knockdown. (A) Immunoblots showing decreased levels of MOF. (B,C) Relative amounts of MOF and H4K16ac evaluated by immunoblotting. The band intensities of MOF (B) and H4K16ac (C) are normalized against those of GAPDH and histone H4, respectively, and further normalized using Con. Plots show between two and six biologically independent experiments (mean±s.e.m., with individual data points). (D) Fluorescence intensity of H4K16ac in MOF knockdown cells (mean±s.e.m.; *n*=3–5 independent experiments indicated by different colors; ≥115 cells were analyzed in each experiment). *P<0.05; **P<0.01; ***P<0.001 (one-way ANOVA test with Tukey test as the post hoc analysis). (E–H) Accumulation kinetics of γ-H2AX. Means±s.e.m. with the total number of cells (indicated as *n*) from between two and four replicates are shown. (E) 11-4 cells with MOF-KD and Con (*P*=0.725 at 105 s, *P*=0.100 at 210 s, and *P*=0.419 at 315 s). (F) 11-4 cells in 2.5 μM NU7441 with MOF-KD and Con (*P*=0.258 at 105 s, *P*=0.636 at 210 s, and *P*=0.235 at 315 s). (G) AT5BIVA cells with MOF-KD and Con (*P*=0.408 at 105 s, *P*=0.062 at 210 s, and *P*=0.100 at 315 s). (H) AT5BIVA cells in 2.5 μM NU7441 with MOF-KD and Con. (I,J) Accumulation kinetics of ATM in AT5BIVA cells that express EGFP–ATM (*P*=0.275 at 105 s, *P*=0.299 at 210 s, and *P*=0.937 at 315 s). (I) Cells without NU7441 with MOF-KD and Con (*P*=0.001 at 105 s, *P*=0.0003 at 210 s, and *P*=0.001 at 315 s). (J) Cells in 2.5 μM NU7441 with MOF-KD and Con (*P*<0.0001 at 105, 210, and 315 s). ***P<0.001 (unpaired, two-tailed Student's *t*-test). w/, with; w/o, without.

We next used a lentivirus-mediated shRNA expression system to investigate the function of MOF in ATM- and/or DNA-PK-mediated H2AX phosphorylation, by knocking down MOF in AT5BIVA cells, 11-4 cells and AT5BIVA cells expressing EGFP–ATM. Immunoblotting showed that MOF-specific shRNA expression lowered MOF and H4K16ac levels to 10–20% and 20–40%, respectively, relative to the expression of scramble shRNA control (Fig. 3A–C). Immunofluorescence confirmed the decrease in H4K16ac upon MOF-specific shRNA expression (Fig. 3D; Fig. S5A–C) and showed that γ-H2AX was still formed by ETP treatment for 20 min in MOF-knockdown cells (Fig. S5A–C). Given that MOF knockdown increased the proportion of apoptotic cells from 1.5% to 18.7% in 11-4 cells and 0.5% to 9.3% in AT5BIVA cells (Fig. S5D) (Li et al., 2012; Thomas et al., 2008), we analyzed cells that showed normal nuclear shape regardless of the cell cycle phase with a laser irradiation assay. MOF knockdown subtly affected the cell cycle progression, increasing the proportion of cell in the G2 and G1 fractions in 11-4 and AT5BIVA cells, respectively, but the majority of cells (47–63%) were in S phase in all cell types (Fig. S5E,F). In both 11-4 and AT5BIVA cells, γ-H2AX Fab kinetics were similar in the MOF knockdown and the scrambled shRNA control cells, both in the absence and presence of the DNA-PK inhibitor NU7441 (Fig. 3E–H), suggesting that MOF does not affect the immediate γ-H2AX formation through ATM and DNA-PK in response to DNA damage. However, the accumulation of EGFP–ATM in the irradiated area was significantly reduced in MOF-knockdown cells compared to in the scrambled control, both without and with NU7441 (Fig. 3I,J). These results imply that MOF facilitates ATM accumulation at damage sites, in agreement with MOF being a regulator of ATM (Gupta et al., 2005); however, the reduced amount of accumulated ATM upon MOF knockdown could still mediate γ-H2AX formation in the early response.

### Mobility of ATM and Ku80, a subunit of DNA-PK, in living cells

**Fig. 4.**
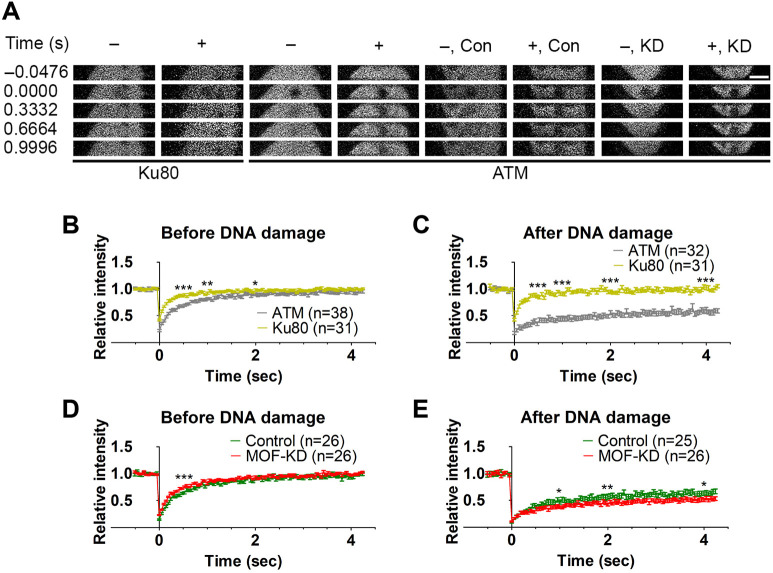
**MOF does not affect ATM binding to the DNA-damaged region.** FRAP using a 488-nm laser was performed for EGFP–ATM in AT5BIVA and EGFP–Ku80 in 11-4 cells. (A) Example images. –, without DNA damage induction; +, with DNA damage induction by 405-nm laser irradiation; Con, control-scramble shRNA expression; KD, MOF knockdown. Scale bar: 5 μm. (B–E) FRAP recovery curves (mean±s.e.m. with the total number of cells, indicated as *n*, from two or three replicates) without (B and D) and with laser irradiation (C and E). (B) EGFP–ATM and EGFP–Ku80 (*P*<0.0001 at 0.5 s, *P*=0.001 at 1 s, *P*=0.027 at 2 s, and *P*=0.800 at 4 s). (C) EGFP–ATM and EGFP–Ku80 in irradiated areas (*P*<0.0001 at 0.5, 1, 2, and 4 s). (D) EGFP–ATM with MOF-KD and Con (*P*=0.0004 at 0.5 s, *P*=0.788 at 1.0 s, *P*=0.243 at 2 s, and *P*=0.225 at 4 s). (E) EGFP–ATM in irradiated areas with MOF-KD and Con (*P*=0.424 at 0.5 s, *P*=0.047 at 1.00 s, *P*=0.007 at 2 s, and *P*=0.021 at 4 s). *P<0.05; **P<0.01; ***P<0.001; ns, not significant (unpaired, two-tailed Student's *t*-test).

We next investigated the mobility of ATM and Ku80, a subunit of DNA-PK, and their response to DNA damage, using FRAP with a 488-nm laser, which does not induce DSBs as does a 405-nm laser (Arimura et al., 2013; Muster et al., 2017). Without DNA damage, EGFP–ATM levels recovered in a few seconds after bleaching. EGFP–Ku80 recovered within 0.5 s, much faster than EGFP–ATM (Fig. 4A,B). In the area with DSBs that were induced by 405-nm laser irradiation, EGFP–ATM recovery became much slower, whereas the recovery kinetics of EGFP–Ku80 remained unchanged (Fig. 4A,C). These results suggest that EGFP–ATM repeatedly binds to and dissociates from chromatin and, when DSBs are induced, EGFP–ATM binds more stably to chromatin. In contrast, EGFP–Ku80 appears to diffuse almost freely in the nucleus. The finding of little or no change in EGFP–Ku80 kinetics in DSBs can be explained by the transient binding of DNA-PK to damaged chromatin and/or if a tiny fraction of DNA-PK is bound to damaged chromatin. In fact, EGFP–Ku80 was not enriched in irradiated areas under the conditions used (see below for the results with more damage).

We also investigated whether or not MOF knockdown affects ATM mobility (Fig. 4A,D,E). MOF knockdown had little effect on the recovery kinetics without or with DNA damage (Fig. 4A,D,E). Thus, the residence time of EGFP–ATM on both undamaged and damaged chromatin does not depend on MOF and H4K16ac, whereas the accumulation of ATM at damaged chromatin appears to be facilitated by MOF (Fig. 3I).

### ATM, but not DNA-PK, binds to chromatin in permeabilized cells

**Fig. 5.**
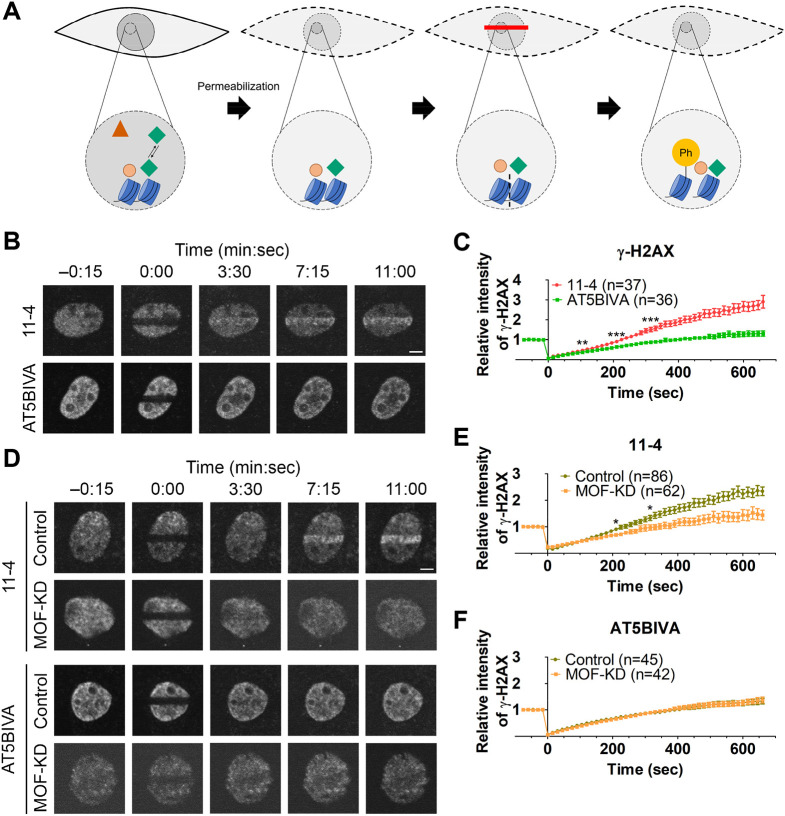
**H2AX phosphorylation activity in permeabilized cells.** (A) Schematic diagram of cell permeabilization and H2AX phosphorylation. Within the intact cells, some proteins (indicated as the red triangle) diffuse freely and others transiently bind to the chromatin (first panel). When cells are permeabilized with 0.1% Triton X-100, only proteins that are not bound to chromatin are extracted (second panel). After DSBs are induced by laser irradiation, histone H2AX becomes phosphorylated if a kinase remains on chromatin in permeabilized cells (third and fourth panels). The γ-H2AX in permeabilized cells can be detected by the accumulation of dye-labeled specific Fab. (B,C) 11-4 and AT5BIVA cells were permeabilized and laser-irradiated in the presence of Cy5-conjugated γ-H2AX Fab. Time-lapse images (B) and accumulation kinetics of γ-H2AX at the irradiated areas (C) are shown (*P*=0.004 at 105 s, and *P*<0.0001 at 210 s and 315 s). (D–F) MOF was knocked down in 11-4 and AT5BIVA cells before permeabilization and laser irradiation. (D) Time-lapse images. (E,F) Accumulation of γ-H2AX at the irradiated areas. (E) 11-4 cells (*P*=0.780 at 105 s, *P*=0.012 at 210 s, and *P*=0.011 at 315 s). (F) AT5BIVA (*P*=0.073 at 105 s, *P*=0.457 at 210 s, and *P*=0.956 at 315 s). Means±s.e.m. with the number of cells (indicated as *n*) from two or three replicates are shown. *P<0.05; **P<0.01; ***P<0.001; ns, not significant (unpaired, two-tailed Student's *t*-test). Scale bars: 5 μm.

To further analyze the different dynamics of ATM and DNA-PK and the relevance to H2AX phosphorylation, we used a permeabilized cell system. When cells are permeabilized with a non-ionic detergent, such as Triton X-100, freely diffusible proteins are extracted, whereas chromatin-bound proteins remain (Jackson and Cook, 1985; Kimura et al., 2006; Nickerson et al., 1997). Permeabilized 11-4 and AT5BIVA cells were incubated with γ-H2AX Fab, and DNA damage was then induced by laser irradiation (Fig. 5A). Accumulation of γ-H2AX Fab in the damaged area was observed in permeabilized 11-4 cells, although it was much slower than that in intact cells (Fig. 5B,C). By contrast, γ-H2AX Fab did not accumulate in permeabilized AT5BIVA cells (Fig. 5B,C). These results suggest that ATM transiently binds to chromatin so that a chromatin-bound fraction remains during permeabilization, whereas DNA-PK freely diffuses without DNA damage and is mostly extracted during permeabilization (Fig. 5A). Immunostaining and EGFP fluorescence indeed confirmed that, in permeabilized cells, a fraction of ATM remained in the nucleus whereas Ku80 was largely extracted (Fig. S6A,B). Thus, in permeabilized cells, only ATM-proficient cells contain H2AX phosphorylation activity in response to laser-induced DNA damage (Fig. 5A). Under MOF knockdown, the accumulation of γ-H2AX Fab was significantly impaired, particularly at the later time points, in 11-4 cells but was unchanged in AT5BIVA cells (Fig. 5D–F), suggesting that MOF has a role in ATM functioning. These data differ from the observation in living cells in which MOF knockdown resulted in little or no effect on γ-H2AX accumulation kinetics (Fig. 3E–H). As proteins dissociated from chromatin can diffuse out from the nucleus in permeabilized cells, the effect of the reduced ATM binding rate upon MOF knockdown might become apparent in permeabilized cells.

### Effects of massive DSBs induced by laser irradiation in sensitized cells

**Fig. 6.**
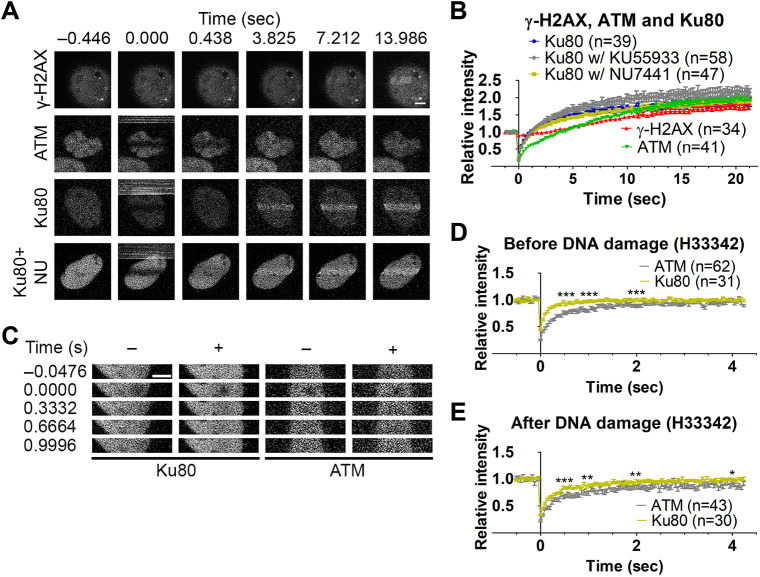
**Effects of massive DSBs induced by Hoechst 33342 sensitization following laser irradiation.** (A) DSBs were induced by irradiating Hoechst 33342-sensitized cells. Time-lapse images were acquired for γ-H2AX Fab and EGFP–Ku80 (with or without 2.5 μM NU7441) in 11-4 cells, and EGFP–ATM in AT5BIVA cells. (B) Accumulation of γ-H2AX and Ku80 in untreated cells, and Ku80 with 2.5 μM NU7441, Ku80 with 5 μM KU55933, and of EGFP–ATM (ATM) in irradiated areas in Hoechst 33342-sensitized cells (mean±s.e.m. with the total number of cells indicated as *n* from two replicates). (C–E) FRAP with a 488-nm laser without and with DSBs. *P*-values for Ku80 with NU7441 or KU55933 (vs control Ku80) at 0.5, 1, 2, and 4 s were all >0.05. (C) Time-lapse images of EGFP–Ku80 and EGFP–ATM before and after bleaching. (D) Fluorescence recovery without DSBs (*P*<0.0001 at 0.5 s, 1 s, and 2 s, and *P*=0.835 at 4 s). (E) Fluorescence recovery with DSBs (*P=*0.0003 at 0.5 s, *P*=0.002 at 1 s, *P*=0.005 at 2 s, and *P*=0.049 at 4 s). *P<0.05; **P<0.01; ***P<0.001; ns, not significant (unpaired, two-tailed Student's *t*-test). w/, with; w/o, without. Scale bars: 5 μm.

Finally, we examined the kinetics of γ-H2AX, EGFP–ATM and EGFP–Ku80 using Hoechst 33342-sensitized cells in which enhanced DNA damage can be induced by laser irradiation (Bekker-Jensen et al., 2006). Within several seconds, γ-H2AX accumulated in irradiated areas in sensitized AT5BIVA cells expressing EGFP–ATM and was then soon enriched in unirradiated areas in the nucleus (Fig. S7A). Unlike previous conditions in unsensitized cells used in this study, EGFP–Ku80 accumulated in laser-irradiated areas, and its accumulation was more rapid than accumulation of γ-H2AX in 11-4 cells and EGFP–ATM in AT5BIVA cells (Fig. 6A,B; Fig. S7B). These data are consistent with the view that abundant and diffused Ku80 rapidly recognizes broken DNA ends, and that DNA-PK initiates H2AX phosphorylation (Davis et al., 2010; Kochan et al., 2017). Treatment with the DNA-PK inhibitor NU7441 did not affect the dynamics of EGFP–Ku80 (Fig. 6B), suggesting that Ku80 accumulation in the damaged region does not depend on the kinase activity of DNA-PK. By contrast, EGFP–Ku80 accumulated slightly more, albeit not significantly, in cells treated with the ATM inhibitor KU55933 (Fig. 6B), which might explain the rapid γ-H2AX accumulation (Fig. 2F). The mobility of EGFP–Ku80 determined by FRAP in sensitized cells became slightly slower after laser irradiation (Fig. 6C–E), suggesting that the residence time on damaged chromatin is shorter for DNA-PK than for ATM, which is in good agreement with a previous report (Davis et al., 2010).

## DISCUSSION

**Fig. 7.**
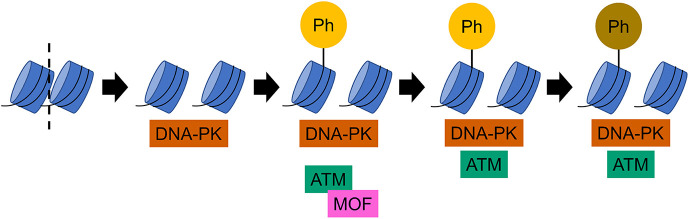
**Model of early γ-H2AX dynamics in response to DSB.** After a DSB, DNA-PK, which diffuses throughout the nucleus, binds to DNA ends and phosphorylates histone H2AX. ATM also phosphorylates H2AX and other proteins, assisted by MOF, although accumulation and activation at damaged sites is slower for ATM than for DNA-PK.

ATM is known as a major kinase that can phosphorylate the histone H2A variant H2AX, to give γ-H2AX, in response to DSBs (Burma et al., 2001; Caron et al., 2015), but the mechanism by which γ-H2AX is formed immediately after DSBs is not fully understood. In this study, we employed Fab-based endogenous modification labeling (Hayashi-Takanaka et al., 2011) to detect the rapid formation of γ-H2AX in response to DNA damage induced by laser irradiation. In both ATM-proficient and -deficient cells, γ-H2AX accumulated immediately after irradiation and reached a broad peak at ∼100–200 s, before gradually decreasing, which might be associated with progression of DNA repair (Bouquet et al., 2006; MacPhail et al., 2003; Mah et al., 2010). Although γ-H2AX accumulation appears to be slightly higher in ATM-proficient cells than ATM-deficient cells, ATM does not appear to have a major role in the immediate γ-H2AX formation upon DNA damage (Fig. 7), in contrast to the later responses in which ATM has a critical role (Caron et al., 2015; Kühne et al., 2004; Löbrich and Jeggo, 2005; Loucas and Cornforth, 2004; Riballo et al., 2004; Stiff et al., 2004). ATM can amplify the DNA damage signal that is initially generated via DNA-PK (Lu et al., 2019), and might be critical for heterochromatin repair that requires a longer time (Goodarzi et al., 2008). Inhibition of DNA-PK using NU7441 altered the kinetics of γ-H2AX leading to it continually accumulating for ≤400–500 s in ATM-proficient cells, but accumulation was drastically delayed in ATM-deficient cells. These results support the critical role of DNA-PK in the immediate DNA damage response (Fig. 7) (Caron et al., 2015; Lu et al., 2019; Riballo et al., 2004), including γ-H2AX phosphorylation (Liu et al., 2019). DNA-PK has also been shown to function in chromatin decompaction and initiation of the DSB response (Lu et al., 2019). Here, we demonstrated that the Ku80 subunit of DNA-PK diffuses freely in the nucleus. It is likely that as soon as DSBs are induced, DNA-PK binds to DNA ends and phosphorylates H2AX more rapidly than ATM, which transiently binds to chromatin in the steady state (Aleksandrov et al., 2018; Davis et al., 2010; Kochan et al., 2017). Once γ-H2AX is formed, it facilitates the binding of ATM to phosphorylate nearby H2AX and DNA repair proteins, such as NBS1 (also known as NBN) (Lim et al., 2000) and p53 (Banin et al., 1998). Thus, the distinct binding and activation mechanisms of ATM and DNA-PK might contribute to the DSB response through overlapping functions in H2AX phosphorylation.

In the absence of DNA-PK activity, γ-H2AX is still formed via ATM, which can bind to both chromatin and DNA repair machinery (Callén et al., 2009). ATM is known to become hyperactivated upon DNA-PK inhibition (Finzel et al., 2016). The slowed and prolonged γ-H2AX accumulation in cells in which DNA-PK activity is inhibited might be caused by this ATM hyperactivation, which later affects the downstream responses of the DNA damage pathway, including the p53 pulse (Finzel et al., 2016; Sun et al., 2017). Despite the fact the EGFP–ATM was much more accumulated during the early response to DNA damage in the presence of DNA-PK inhibitor compared to in the untreated control, γ-H2AX accumulation was rather delayed. This suggests that the amount of ATM on damage sites is not necessarily correlated with its phosphorylation activity. This notion is also supported by our MOF knockdown experiments where ATM accumulates at a lower level but γ-H2AX accumulation remains the same upon knockdown. Hence, ATM activity appears to be robustly regulated to compensate for the variation in the amount of accumulation, although the molecular mechanism needs to be elucidated.

The DSB repair pathway choice depends on the cell cycle (Her and Bunting, 2018; Karanam et al., 2012). NHEJ occurs throughout the cell cycle but preferentially functions during the G1 phase (Lieber et al., 2003; Mao et al., 2008b), whereas HR, which uses the sister chromatid as a template for repair can be used in the S and G2 phases (Kadyk and Hartwell, 1992). Although the difference in the DNA repair pathway might affect the dynamics of γ-H2AX (Shrivastav et al., 2008), the immediate early accumulation kinetics were similar in all cell cycle phases with or without ATM. Taken together with the critical role of DNA-PK, this observation is consistent with the idea that the preferred pathway in mammalian cells is NHEJ for faster and more efficient repair processes (Mao et al., 2008a,b).

Our results show that using γ-H2AX-specific Fabs is a powerful tool to study the early dynamics of DSBs. However, given that using the ATM inhibitor does not phenocopy the ATM-mutated cells (Choi et al., 2010), DNA-PK inhibitor treatment might also not phenocopy the DNA-PK-deficient cells. Therefore, future studies should investigate whether the delay in the accumulation of γ-H2AX is still observed in the DNA-PK-deficient cells or not. In addition, as the dynamics of p53 differs in different cell lines (Stewart–Ornstein and Lahav, 2017), future studies should address whether the γ-H2AX dynamics depends on p53 or vice versa. In summary, our results confirm that ATM is dispensable for histone H2A phosphorylation in the immediate early response to DSBs, and support the significance of DNA-PK in this process.

## MATERIALS AND METHODS

### Cell culture

AT5BIVA, an SV40-transformed AT fibroblast cell line, and 11-4 cells, which are AT5BIVA cells with the addition of chromosome 11 to restore ATM, were gifts from Satoshi Tashiro (Hiroshima University, Japan) (Sun et al., 2010). The expression levels of ATM in these cells were confirmed by western blotting. HEK293T cells were obtained from Kei Fujinaga (Sapporo Medical University, Japan) and its ability to produce lentivirus was confirmed by infection assays. AT5BIVA, 11-4 and HEK293T cells were routinely cultured in Dulbecco's modified Eagle's medium (DMEM; Nacalai Tesque) supplemented with 10% fetal bovine serum (FBS; Thermo Fisher Scientific) and 1% L-glutamine-penicillin-streptomycin solution (GPS; Sigma-Aldrich) at 37°C under 5% CO_2_. Mycoplasma-free conditions were routinely confirmed by Hoechst 33342 (Nacalai Tesque) staining.

### Plasmids and transfection

Cells were plated in a six-well plate (Thermo Fisher Scientific) 1 day before the transfection of the expression constructs for mCherry-tagged proliferating-cell nuclear antigen (PCNA; Leonhardt et al., 2000) or EGFP-ATM based on PB533A-2 (System Biosciences) with a PiggyBac transposon expression vector (PB210PA-1; System Biosciences), or pEGFP-C1-FLAG-Ku80 (Addgene 46958; Britton et al., 2013) using FuGENE HD Transfection Reagent (Promega) according to the manufacturer's instruction. The EGFP-ATM expression vector was constructed using a plasmid containing ATM cDNA provided by Tsuyoshi Ikura (Kyoto University, Japan) and the entire ATM sequence was verified by sequencing. To obtain a stable cell line, 2 days after the transfection, the cells were incubated in the presence of 1 mg/ml G418 disulfate aqueous solution (Nacalai Tesque) in the complete medium for >1 week. Cells that exhibited mCherry–PCNA or EGFP–ATM fluorescence were sorted using a cell sorter (Sony; SH800) and cultured in fresh medium without G418.

### Lentiviral shRNA infection

HEK293T cells were plated 1 day before transfection with psPax2 (viral packaging plasmid; Addgene 12260), pCMV-VSV-G (viral envelope plasmid; Addgene 8454), and a pLKO.1-based plasmid containing either scrambled sequences or human MOF shRNA (Kapoor-Vazirani et al., 2008, 2011; pCMV-VSV-G and pLKO.1 puromycin; Addgene 8454 and 8453, respectively) using Lipofectamine 3000 (Invitrogen) according to the manufacturer's instruction. The pLKO.1-based plasmids were constructed according to Addgene's protocol. Medium containing lentiviral particles collected 1 day after transfection were filtered through a 0.45-μm filter (Advantec), and then 2 μg/ml polybrene (Sigma-Aldrich) was added. Recipient cells were plated 1 day before the infection and the medium was replaced with a lentiviral-containing medium for 18–24 h. Then, the medium was changed to a fresh medium containing 1 μg/ml puromycin (Thermo Fisher Scientific) for 11-4 cells and 2 μg/ml puromycin for AT5BIVA cells. After 1–2 days of puromycin selection, the medium was replaced with fresh medium.

### Live-cell imaging

Cells were plated on a 35-mm glass-bottom dish with a coverslip (AGC Techno Glass). The next day, fluorescent dye-labeled modification-specific Fab was loaded into cells using glass beads (Hayashi-Takanaka et al., 2011) and the medium was changed to FluoroBrite DMEM (Thermo Fisher Scientific) supplemented with 10% FBS and 1% GPS. The preparation and dye-conjugation of Fabs have been described previously (Kimura and Yamagata, 2015; Yamagata et al., 2019). A glass-bottom dish was set on a heated stage (Tokai Hit) with a CO_2_ control system (Token) on a confocal microscope (FV-1000, Olympus) operated by built-in software (Fluoview ver. 4.2) with a PlanSApo 60× (NA 1.40) oil-immersion objective lens to maintain cells at 37°C under 5% CO_2_. For the laser-irradiation assay, five images were collected using the line-sequential imaging mode (512×512 pixels; pinhole 800 μm; 8× zoom; 2-line Kalman filtration) with three laser lines (0.1–2.0% 488-nm laser transmission; 0.5–10.0% 543-nm laser transmission; and 0.5–5.0% 633-nm laser transmission), then a 26.06×2.25-μm rectangle area was irradiated with 100% 405-nm laser transmission for 3.09 s, and another 45 images were collected using the original settings at 15-s intervals. The time-series images were aligned, and the fluorescence intensities in the irradiated and unirradiated areas were measured using CellProfiler 4.0.7 image analysis software (Stirling et al., 2021). The relative intensity of the irradiated area was calculated by performing double normalization. After background subtraction, the intensity of the irradiated area was divided by that of the nucleus, and then the intensity ratio was divided by the average ratio before irradiation.

For FRAP (Arimura et al., 2013), ten images were collected (2.0% 488-nm laser transmission; 128×24 pixels; pinhole 800 μm; 10× zoom), a 1.6 μm diameter circle area was bleached (100% 488-nm laser transmission; 31 ms), and another 90 images were collected consecutively.

To compare Ku80 dynamics with ATM and γ-H2AX, 11-4 cells that expressed EGFP–Ku80, and AT5BIVA cells that expressed EGFP–ATM were sensitized with Hoechst 33342 (Nacalai Tesque) at 0.8 μM for 1 h before performing the laser microirradiation assay (512×512 pixels).

For overnight observation to study the changes in PCNA distribution at different cell cycle phases, 11-4 and AT5BIVA cells expressing mCherry–PCNA were plated on a 35-mm glass-bottom dish. Cells were observed with a spinning disk confocal microscope (CSU-W1; Yokogawa and Ti-E; Nikon) with a PlanApo VC 100× (NA 1.40) oil-immersion objective lens equipped with an electron-multiplying charge-coupled device (iXon+; Andor) and a 488-nm laser (Nikon; LU-N4) at 37°C under 5% CO_2_. The images were captured with the NIS-Elements analysis software ver. 5.1 (Nikon).

### Inhibitor treatment and immunofluorescence

The inhibitors against ATR (AZ20), ATM (KU55933), and DNA-PK (NU7441) were purchased from Tocris Bioscience and dissolved in dimethyl sulfoxide (DMSO; Nacalai Tesque). To optimize the concentration of each inhibitor, cells plated in EZVIEW™ Glass Bottom Culture Plates LB (24 well; AGC Techno Glass) a day before cells were treated with each inhibitor at 10, 5 and 2.5 μM or with DMSO alone, simultaneously with etoposide (ETP; Sigma-Aldrich) at 20 μg/ml for 1 h at 37°C. The following procedures were performed at room temperature. Cells were fixed with 4% paraformaldehyde (PFA; Electron Microscopy Sciences) in 250 mM HEPES-NaOH (pH 7.4) containing 0.1% Triton X-100 (Nacalai Tesque) for 5 min at room temperature, washed with Ca^2+^- and Mg^2+^-free Dulbecco's phosphate buffered saline (PBS; Fujifilm Wako Chemicals), and permeabilized using 1% Triton X-100 for 20 min with gentle shaking. The cells were washed with PBS, incubated in blocking solution (Blocking-One P; Nacalai Tesque) for 20 min with gentle shaking, and washed with PBS. The cells were stained with anti-γ-H2AX antibody (2 μg/ml; Yamagata et al., 2019) for 1 h with gentle shaking, washed with PBS three times, and stained with goat anti-mouse IgG (H+L) (0.5 μg/ml; Jackson ImmunoResearch) conjugated to Alexa Fluor 488 (Thermo Fisher Scientific) and Hoechst 33342 (0.1 μg/ml; Nacalai Tesque) for 1 h with gentle shaking. The cells were washed with PBS before observation using a wide-field fluorescence microscope (Ti-E; Nikon) under the operation of NIS-Elements version 3.0 (Nikon) with a Plan Apo 40× (NA 0.95) objective lens, an electron-multiplying charge-coupled device (EM-CCD; iXon+; Andor; normal mode; gain ×5.1), an LF488-A filter set (Semrock), and a 75-W Xenon lamp as a light source.

MOF-knockdown cells were plated in an eight-well μ-Slide (ibidi) 1 day before ETP treatment (20 μg/ml) for 20 min at 37°C. The cells were fixed, permeabilized and blocked as above before staining with fluorescence dye-labeled primary antibodies. MOF-knockdown cells that expressed mCherry–PCNA were stained with 2 μg/ml Alexa Fluor 488-conjugated anti-γ-H2AX antibody and 2 μg/ml Cy5-conjugated anti-H4K16ac antibody (Hayashi-Takanaka et al., 2015) overnight at 4°C with gentle shaking. MOF-knockdown AT5BIVA cells that express EGFP–ATM were stained with 2 μg/ml Cy5-conjugated anti-γ-H2AX antibody (Yamagata et al., 2019) and 2 μg/ml Cy3-conjugated anti-H4K16ac antibody (Hayashi-Takanaka et al., 2015) overnight in Can-Get-Signal^®^ Immunostain Immunoreaction Enhancer Solution B (Toyobo). The cells were washed with PBS, stained with Hoechst 33342 (0.1 μg/ml) for 1 h at room temperature with gentle shaking, and washed again with PBS before observation using a spinning-disk confocal microscope (CSU-W1; Yokogawa and Ti-E; Nikon) under the operation of NIS-Elements version 5.1 (Nikon) with a PlanApo 40× (NA 0.95) objective lens, an EM-CCD (iXon+; Andor; EM gain 300; gain ×5.1) and a 405, 488, 561 and 647 laser system (LU-N4; Nikon).

The image analysis was performed using the NIS-elements Analysis software ver. 5.1 (Nikon); nuclear areas were automatically defined by thresholding using Hoechst 33342 signals. The fluorescence intensity of each channel in the individual nucleus was then measured.

### Western blotting

Cell lysates were prepared by collecting the cultured cells by trypsinization, washing with cold PBS (Takara) and resuspension in lysis buffer [150 mM NaCl, 1% Triton X-100, 0.5% sodium deoxycholate (Fujifilm Wako Chemicals), 50 mM Tris-HCl pH 8.0 (Nacalai Tesque)]. The protein concentration was measured by using the Protein Assay BCA Kit (Fujifilm Wako Chemicals) and bovine serum albumin as the standard according to the manufacturer's instructions. Each sample was mixed with a sample-loading buffer [125 mM Tris-HCl pH 6.8, 20% glycerol (Fujifilm Wako Chemicals), 4% sodium dodecyl sulfate (SDS; Fujifilm Wako Chemicals), 0.01% bromophenol blue (Fujifilm Wako Chemicals), and 10% dithiothreitol (Fujifilm Wako Chemicals)], heated at 95°C for 10 min. Then, 5–15 μl of each sample was separated on 7.5% (for ATM and RNA polymerase II) or 15% (for MOF, GAPDH, H4K16ac, and H4) polyacrylamide gels (SuperSep™ Ace, 17 well pre-cast; Fujifilm Wako Chemicals). The proteins on the gels were transferred to FluoroTrans W PVDF Transfer Membranes (Pall; 90 min; 170 mA constant for a 9 cm×9 cm membrane) using EzFastBlot (Atto) as a transfer buffer. The membranes were blocked with Blocking One (Nacalai Tesque) for 30 min with gentle shaking. After washing with TBS-T (20 mM Tris-HCl, pH 8.0, 150 mM NaCl and 0.02% Tween 20), the membranes were incubated with the primary antibody rabbit anti-ATM (1:1000; Abcam; Y170; a gift from Satoshi Tashiro), rabbit anti-MOF/MYST1 (1 μg/ml), mouse anti-H4K16ac (2 μg/ml) (Hayashi-Takanaka et al., 2015), mouse anti-RNA polymerase II CTD (2 μg/ml) (Stasevich et al., 2014), and mouse anti-GAPDH (0.1 μg/ml; Santa Cruz Biotechnology; 6C5) diluted in Can-Get-Signal^®^ Solution 1 (Toyobo) overnight at 4°C. After washing the membranes with TBS-T three times, the membranes were incubated with horseradish peroxide-conjugated goat anti-mouse IgG (H+L) (1:2000; Jackson ImmunoResearch) or goat anti-rabbit IgG (H+L) (1:2000; Jackson ImmunoResearch) diluted in Can-Get-Signal^®^ Solution 2 (Toyobo) for 1 h at room temperature. The membranes were then washed with TBS-T. For chemiluminescence detection using a gel imaging system (LuminoGraph II, Atto), Western Lightning^®^ Plus-ECL (PerkinElmer) and ImmunoStar^®^ LD (Fujifilm Wako Chemicals) were used for RNA polymerase II and other proteins, respectively. For detecting total H4, WB Stripping Solution Strong (Nacalai Tesque) was used to strip the anti-H4K16ac antibody before reprobing with a pan-H4 antibody that binds to H4 regardless of the modification states (2 μg/ml; CMA401; Hayashi-Takanaka et al., 2015). Uncropped gel images are shown in Fig. S8.

### Cell permeabilization

To extract proteins that freely diffuse in cells, the cells were permeabilized as described previously (Kimura et al., 2006). Cells that had been plated on a 35-mm glass-bottom dish 1 day before were chilled on ice, and washed twice with ice-cold PBF [100 mM potassium acetate, 30 mM KCl, 10 mM Na_2_HPO_4_, 1 mM dithiothreitol, 1 mM MgCl_2_, 1 mM adenosine triphosphate (Thermo Fisher Scientific), and 5% Ficoll (Nacalai Tesque)] followed by incubation with ice-cold PBF containing 0.1% Triton X-100 for 5 min. The cells were washed twice with cold PBF and incubated with Cy5-conjugated γ-H2AX Fab and Alexa Fluor 488-conjugated H4K20me2 Fab in PBF for 3–4 h on ice. The laser irradiation assay and observation were performed at 29°C using a confocal microscope (FV-1000), as described above. To compare the levels of endogenous proteins that remained after permeabilization, cells without or with permeabilization were fixed, treated with Triton X-100, and incubated with mouse anti-ATM (0.2 μg/ml; Santa Cruz Biotechnology; G-12) or mouse anti-Ku86 (1 μg/ml; Santa Cruz Biotechnology; B-1) antibody, and then with Cy3-conjugated goat anti-mouse IgG (H+L) (0.5 μg/ml; Jackson ImmunoResearch) and Hoechst 33342, as described above.

### Statistical analysis

For the dynamics of γ-H2AX accumulation, a Student's *t*-test (unpaired, two-tailed) or one-way ANOVA test with Tukey test as the post hoc analysis was performed at 105, 210 and 315 s. For FRAP experiments, the Student's *t*-test (unpaired, two-tailed) was performed at 0.5, 1, 2 and 4 s. For immunofluorescence data, a one-way ANOVA test with Tukey test as the post hoc analysis was performed. IBM SPSS Statistics for Windows, version 22 (IBM Corp.) was used for statistical analysis. Statistical significance is indicated by asterisks (*P<0.05; **P<0.01; ***P<0.001).

## Supplementary Material

10.1242/joces.260698_sup1 Supplementary information
