# Supplementary material for: Live-cell tracking of γ-H2AX kinetics reveals the distinct modes of ATM and DNA-PK in the immediate response to DNA damage
Source: J Cell Sci. 2023 Apr 27;136(8):jcs260698. doi: 10.1242/jcs.260698 (PMC10163350; doi:10.1242/jcs.260698)
Supplement: Supplementary information [file joces-136-260698-s1.pdf]

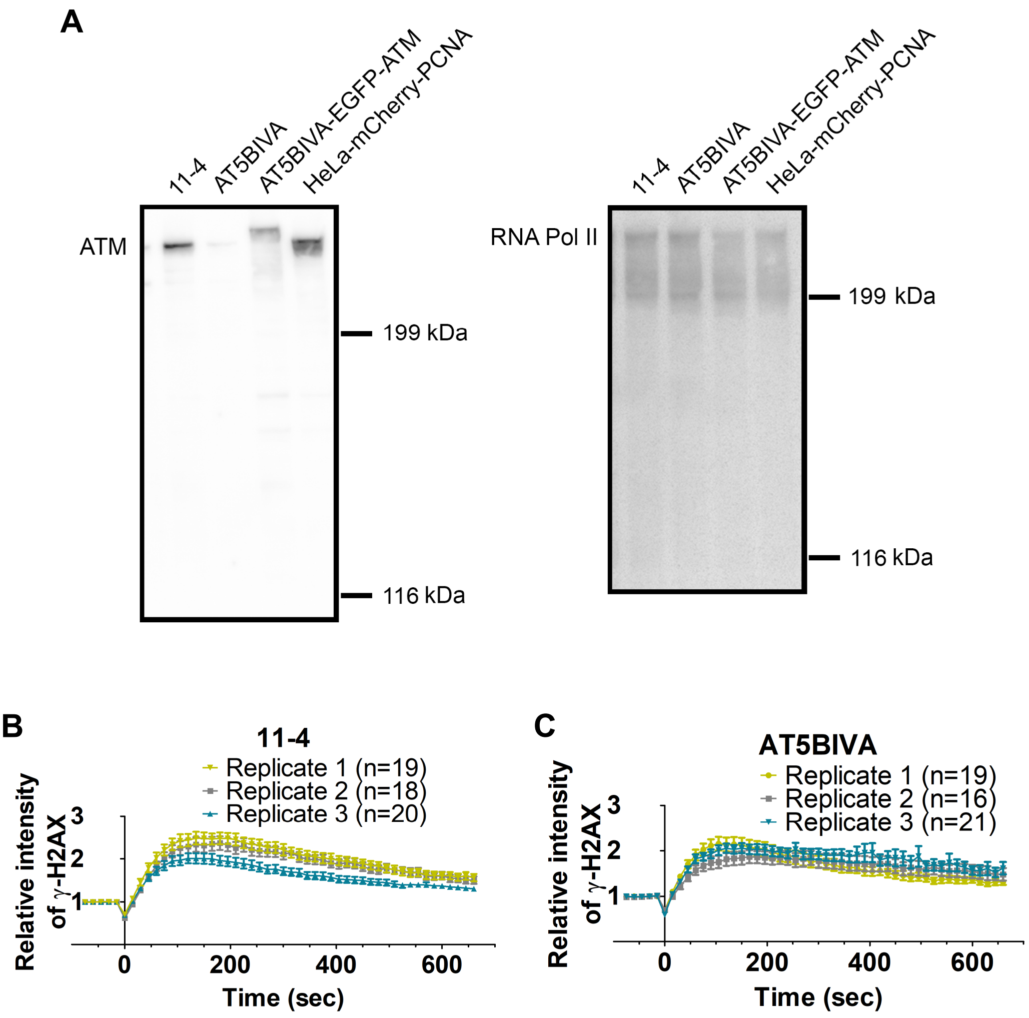

Fig. S1. ATM expression in different cell lines and the early dynamics of γ-H2AX in 11-4 and AT5BIVA cells in each replicate.

(A) Cell lysates were separated on 7.5% SDS-polyacrylamide gels, transferred to membranes, and blotted with antibodies specific for ATM (left) and RNA polymerase II (right). Positions of size standards are indicated on the right. (B and C) Accumulation of γ-H2AX Fab in the laser-irradiated area in each replicate (mean ± SEM with the number of cells, n). The average of all cells was used in Fig. 1C. (B) 11-4 cells. (C) AT5BIVA cells. The curves are similar although there are experiment-to-experiment variations.

Journal of Cell Science • Supplementary information

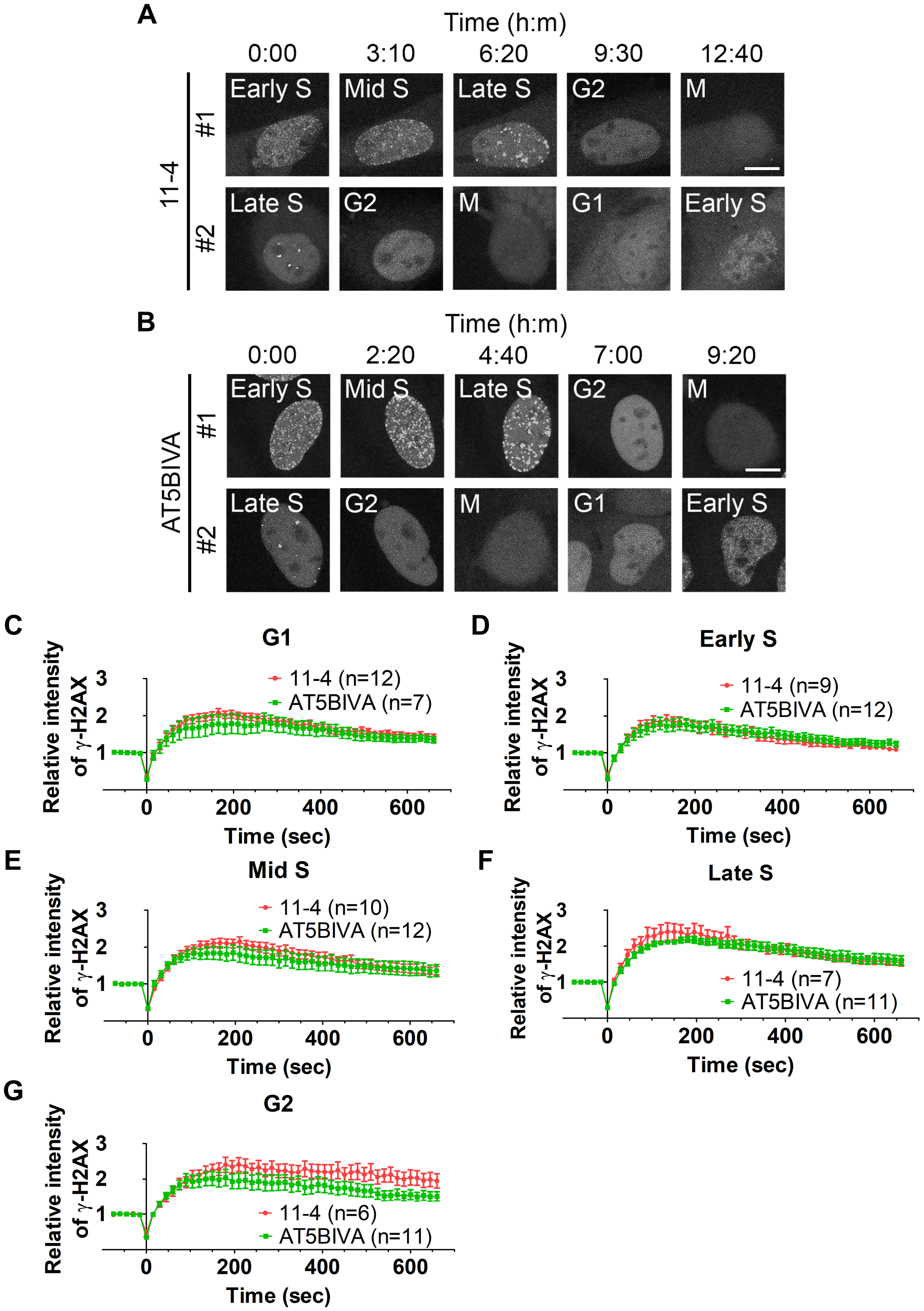

Fig. S2. Accumulation kinetics of γ-H2AX in cells at different cell-cycle phases.

To judge the cell-cycle phase of individual cells, mCherry-PCNA was expressed in 11-4 and AT5BIVA cells. (A and B) Time-lapse images of mCherry-PCNA in 11-4 (A) and AT5BIVA (B) cells. In G1 cells, mCherry-PCNA distributed in both the nucleus and cytoplasm. Numerous foci of mCherry-PCNA are detected in the early-S phase and become bigger and fewer as the cell progresses through the mid- to late-S phase. mCherry-PCNA distributed throughout the nucleus except the nucleoli in the G2 phase. (C–G) Accumulation kinetics of γ-H2AX (mean ± SEM with the number of cells, n, from 2 replicates). (C) G1 (p = 0.442 at 105 s, p = 0.449 at 210 s, and p = 0.552 at 315 s). (D) early-S (p = 0.591 at 105 s, p = 0.859 at 210 s, and p = 0.612 at 315 s). (E) mid-S (p = 0.503 at 105 s, p = 0.183 at 210 s, and p = 0.448 at 315 s). (F) late-S (p = 0.342 at 105 s, p = 0.364 at 210 s, and p = 0.868 at 315 s). (G) G2 (p = 0.584 at 105 s, p = 0.179 at 210 s, and p = 0.329 at 315 s). Scale bar: 10 μm.

Journal of Cell Science • Supplementary information

J. Cell Sci.: doi:10.1242/jcs.260698: Supplementary information

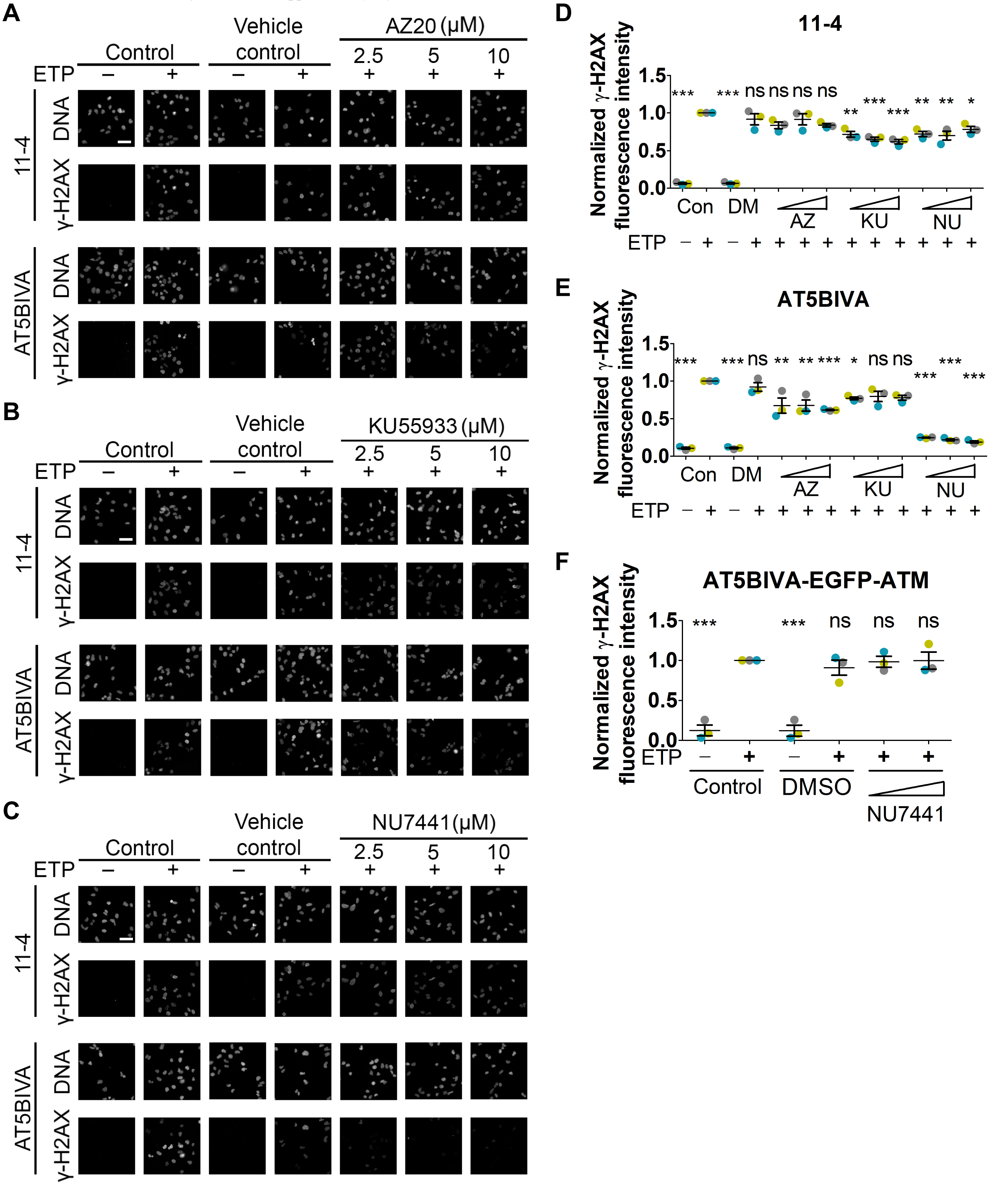

Fig. S3. Optimizing the concentration of ATR, ATM, and DNA-PK inhibitor.

(A–E) Treatment of 11-4 and AT5BIVA cells with AZ20, an ATR inhibitor (A), KU55933, an ATM inhibitor (B), and NU7441, a DNA-PK inhibitor (C), at 0, 2.5, 5, and 10 μM without or with ETP at 20 μg/mL for 1 h. The cells were fixed and stained with γ-H2AX-specific antibody followed by Alexa Fluor 488-conjugated anti-mouse antibody. DNA was stained with Hoechst 33342. (A–C) Fluorescence images. (D and E) The normalized γ-H2AX fluorescence intensities in individual nuclei of 11-4 (D) and AT5BIVA (E) cells were measured in ≥60 nuclei per experiment and the mean intensities were obtained. Means ± SEM from three independent experiments are plotted with each data point. (F) AT5BIVA cells expressing EGFP-ATM were treated with NU7441 at 2.5 and 5 μM simultaneously with ETP at 20 μg/mL for 1 h. The cells were fixed and stained with γ-H2AX-specific antibody followed by Cy3-conjugated anti-mouse antibody. Means ± SEM are plotted with each data point as in (D) and (E). Single, double, and triple asterisks (*, **, and ***) indicate *p* values < 0.05, < 0.01, and < 0.001, respectively, by comparing the Control with ETP+. ns indicates "not-significant (*p* value ≥ 0.05)". Scale bar: 50 μm.

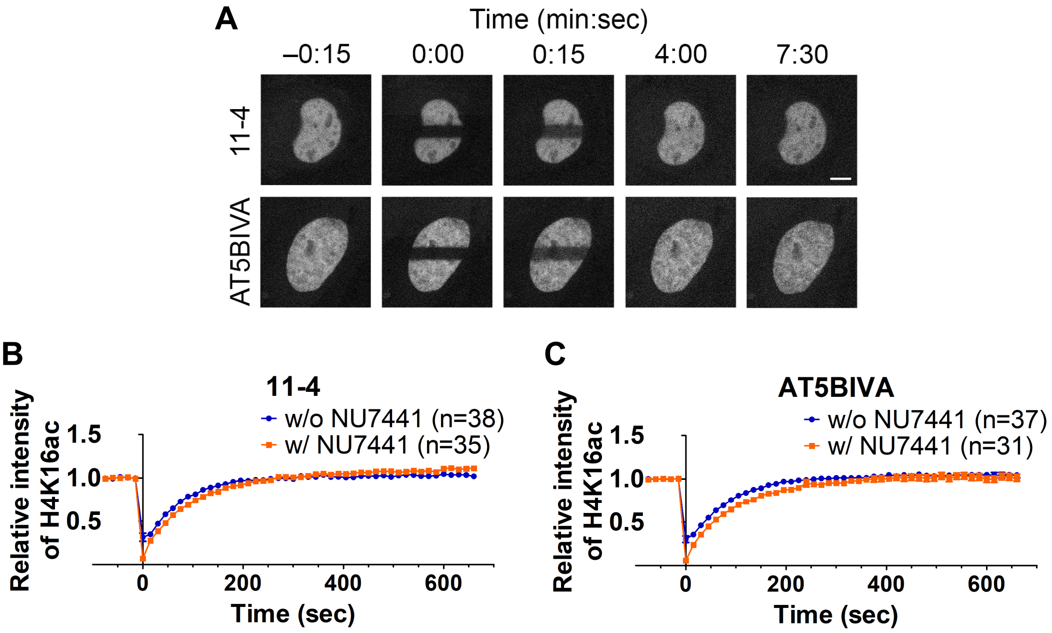

Fig. S4. H4K16ac in 11-4 and AT5BIVA cells.

Loading of 11-4 and AT5BIVA cells with H4K16ac Fab followed by treatment without or with 2.5 μM of NU7441 for ≥1 h before inducing DNA damage by laser irradiation. The Fab intensities in the irradiated areas were measured and plotted (mean ± SEM, with the total number of cells, n, from 2 replicates). The fluorescence intensity recovered to the original level after DNA damage in both cells without or with NU7411, indicating that H4K16ac level did not change during the time scale analyzed. Scale bar: 5 μm.

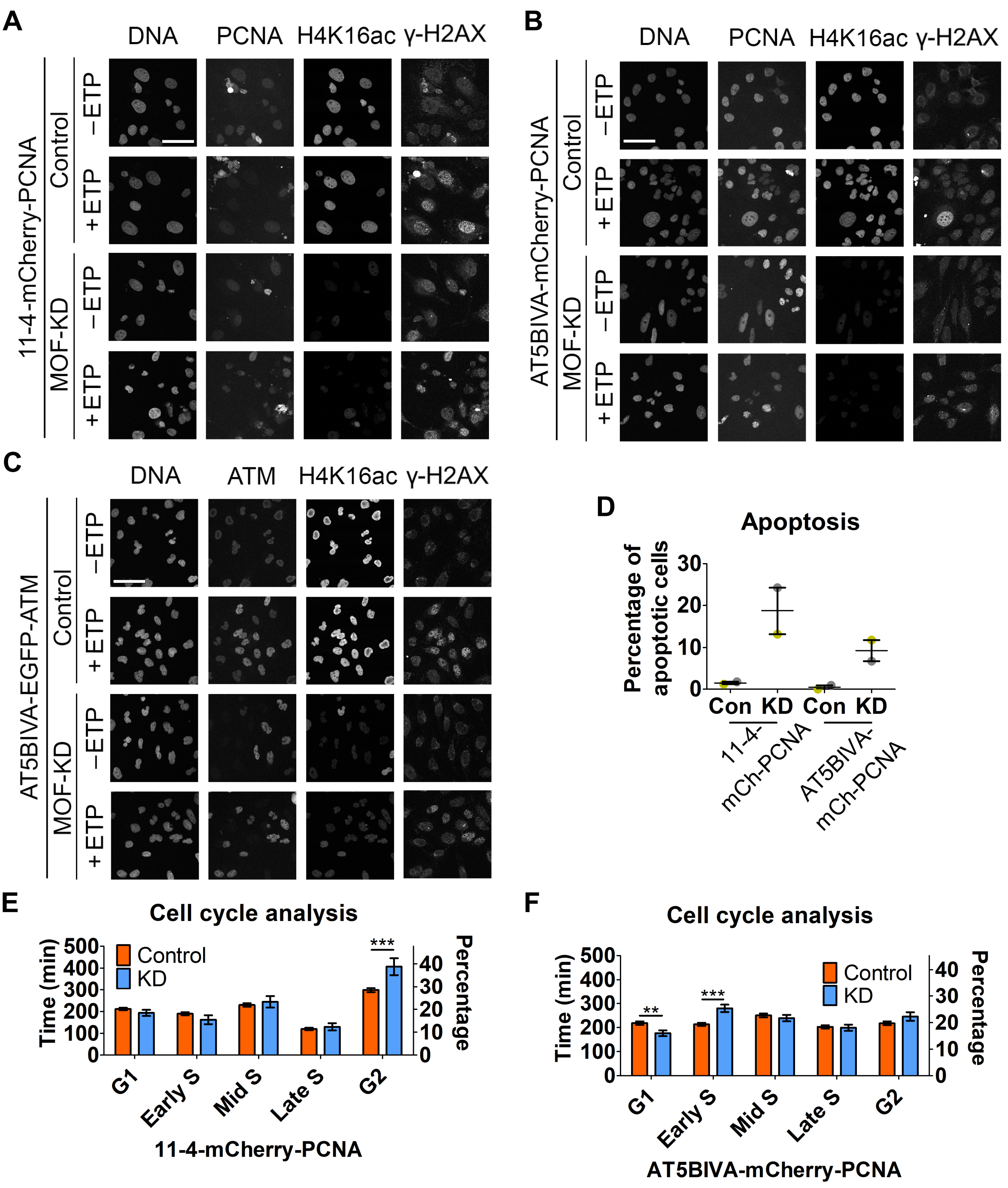

Fig. S5. Characteristics of MOF-knockdown cells.

MOF was knocked down in 11-4 cells expressing mCherry-PCNA (A), AT5BIVA cells expressing mCherry-PCNA (B), and AT5BIVA cells expressing EGFP-ATM (C). The cells were treated with ETP at 20 μg/mL for 20 min to induce DNA damage, and fixed. The 11-4 and AT5BIVA cells expressing mCherry-PCNA were stained with Alexa Fluor 488-labeled γ-H2AX and Cy5-labeled H4K16ac antibodies. The AT5BIVA cells expressing EGFP-ATM were stained with Cy3-labeled H4K16ac and Cy5-labeled γ-H2AX antibodies. (D) The percentage of apoptotic cells in MOF knockdown cells of 11-4 cells expressing mCherry-PCNA and AT5BIVA cells expressing mCherry-PCNA. (E and F) The duration of each cell cycle phase was measured by time-lapse imaging based on PCNA distribution (left y-axis) for 11-4 cells expressing mCherry-PCNA (E) and AT5BIVA cells expressing mCherry-PCNA (F). The percentage to the total cell cycle duration is indicated on the right axis. Double and triple asterisks (** and ***) indicate p values < 0.01 and < 0.001, respectively. Scale bar: 50 μm.

Journal of Cell Science • Supplementary information

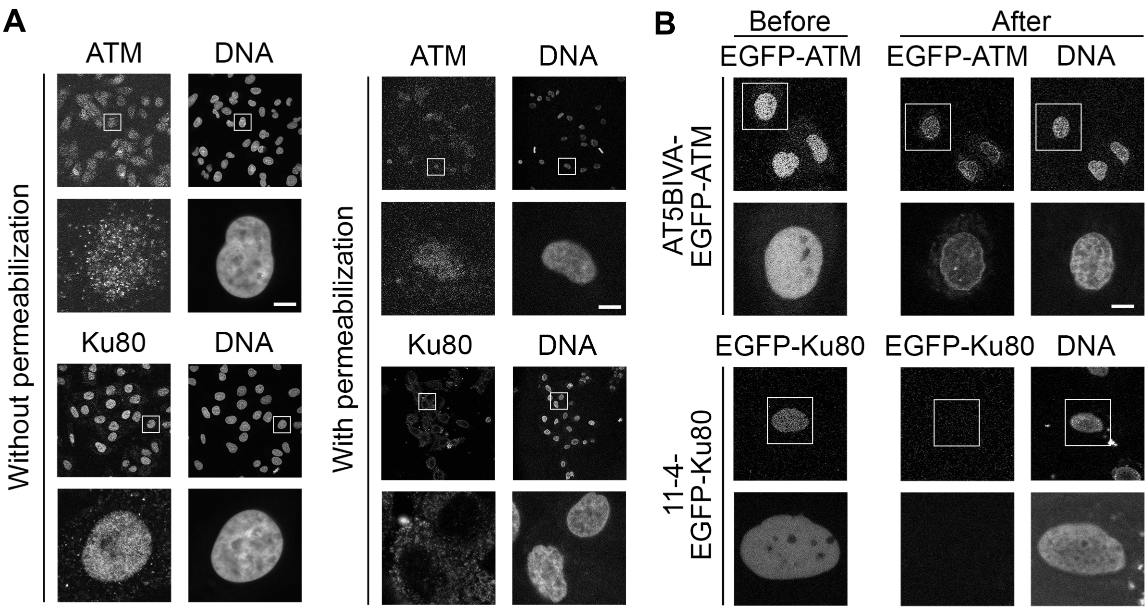

Fig. S6. Localization of ATM and Ku80 in permeabilized cells

(A) Immunofluorescence of ATM and Ku80 in 11-4 cells with and without permeabilization before fixation. ATM, but not Ku80, was detected in permeabilized cells using their specific antibodies.(B) EGFP-ATM was stably expressed in AT5BIVA and EGFP-Ku80 was transiently expressed in 11-4 before and after permeabilization. Cells were plated on a glass-bottom dish with a grid. After collecting fluorescence images, cells were permeabilized and fixed, before acquiring the images for the same cells. Some EGFP-ATM signals were retained after permeabilization whereas no detectable EGFP-Ku80 signals were observed. Magnified views in boxed areas are shown at the bottom row. Scale bars: 5 μm.

Journal of Cell Science • Supplementary information

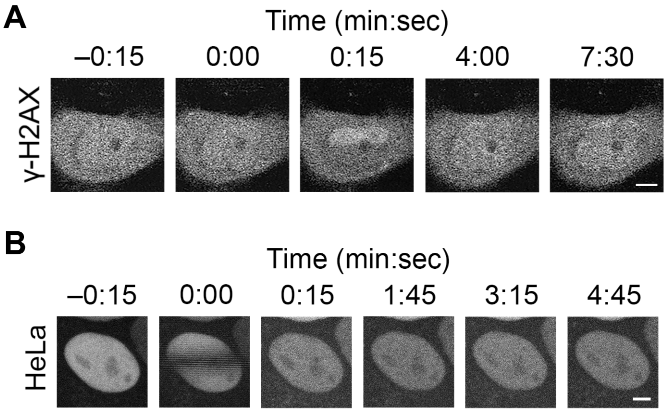

Fig. S7. EGFP-Ku80 and EGFP-ATM in Hoechst 33342-sensitized cells.

(A) Accumulation of γ-H2AX in AT5BIVA cells expressing EGFP-ATM that were sensitized with 0.8 μM Hoechst 33342 for 1 h before laser irradiation.(B) Accumulation of EGFP-Ku80 in HeLa cells without Hoechst 33342 sensitization. Scale bars: 5 μm.

Journal of Cell Science • Supplementary information

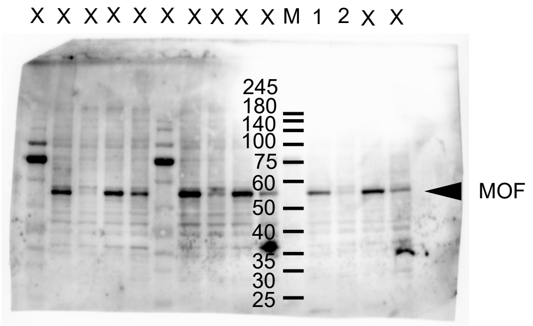

Fig. 3A
M: Marker (kDa)
1: 11-4-mCherry-PCNA (Control
2: 11-4-mCherry-PCNA (MOF-KD)

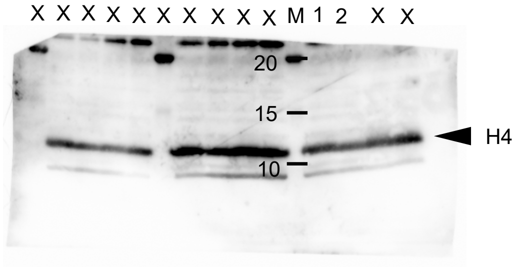

Fig. 3A
M: Marker (kDa)
1: 11-4-mCherry-PCNA (Control
2: 11-4-mCherry-PCNA (MOF-KD)

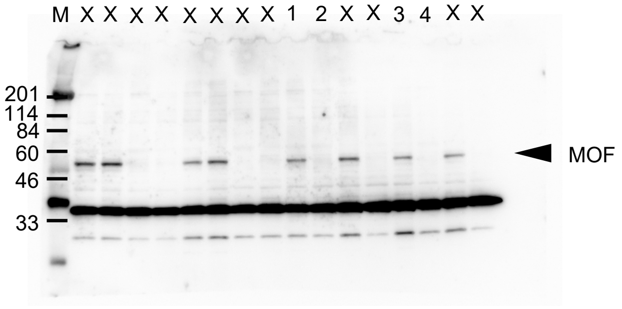

Fig. 3A
M: Marker (kDa)
1: AT5BIVA-mCherry-PCNA (Control)
2: AT5BIVA-mCherry-PCNA (MOF-KD)
3: AT5BIVA-EGFP-ATM (Control)
4: AT5BIVA-EGFP-ATM (MOF-KD)

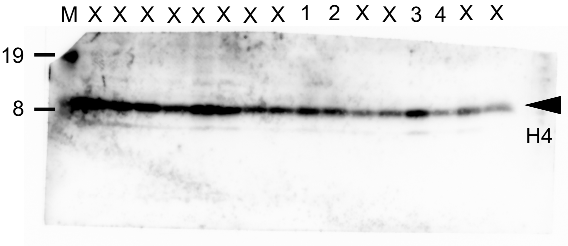

Fig. 3A
M: Marker (kDa)
1: AT5BIVA-mCherry-PCNA (Control)
2: AT5BIVA-mCherry-PCNA (MOF-KD)
3: AT5BIVA-EGFP-ATM (Control)
4: AT5BIVA-EGFP-ATM (MOF-KD)

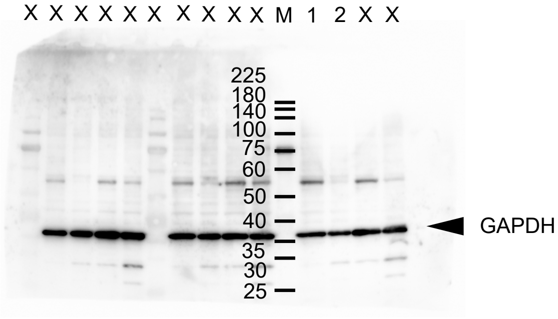

Fig. 3A
M: Marker (kDa)
1: 11-4-mCherry-PCNA (Control
2: 11-4-mCherry-PCNA (MOF-KD)

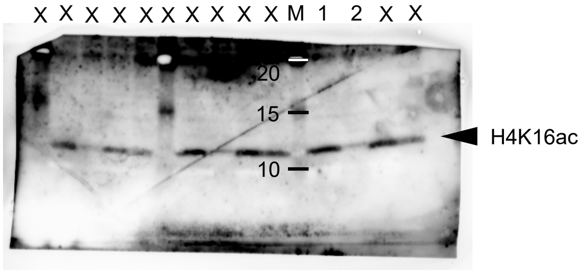

Fig. 3A
M: Marker (kDa)
1: 11-4-mCherry-PCNA (Control
2: 11-4-mCherry-PCNA (MOF-KD)

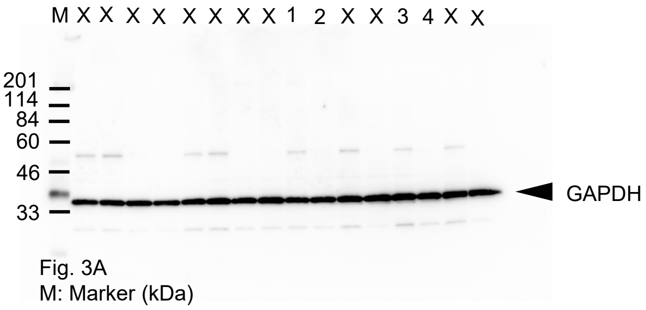

Fig. 3A
M: Marker (kDa)
1: AT5BIVA-mCherry-PCNA (Control)
2: AT5BIVA-mCherry-PCNA (MOF-KD)
3: AT5BIVA-EGFP-ATM (Control)
4: AT5BIVA-EGFP-ATM (MOF-KD)

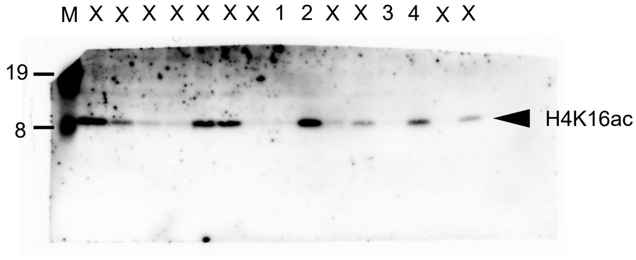

Fig. 3A
M: Marker (kDa)
1: AT5BIVA-mCherry-PCNA (Control)
2: AT5BIVA-mCherry-PCNA (MOF-KD)
3: AT5BIVA-EGFP-ATM (Control)
4: AT5BIVA-EGFP-ATM (MOF-KD)

Fig. S8. 1. Blot transparency (uncropped gels).

Journal of Cell Science • Supplementary information

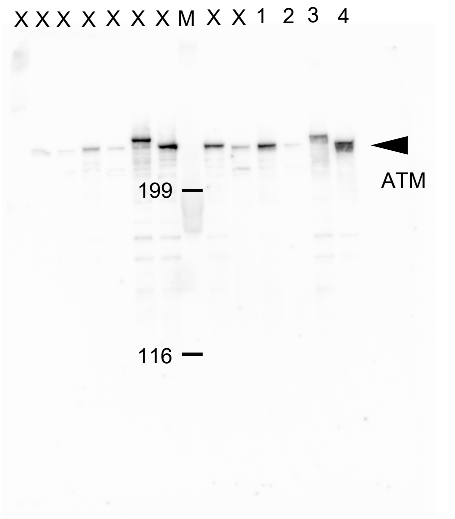

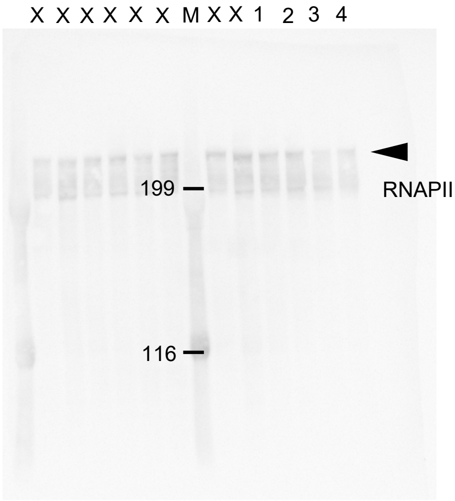

Fig. S8. 2. Blot transparency (uncropped gels).

Journal of Cell Science • Supplementary information
