# Supplementary material for: Live-cell tracking of γ-H2AX kinetics reveals the distinct modes of ATM and DNA-PK in the immediate response to DNA damage
Source: J Cell Sci. 2023 Apr 27;136(8):jcs260698. doi: 10.1242/jcs.260698 (PMC10163350; doi:10.1242/jcs.260698)
Supplement: Supplementary file 1 [file joces-136-260698_review_history.pdf]

Journal of Cell Science

Live-cell tracking of γ-H2AX kinetics reveals the distinct modes ofATM and DNA-PK in the immediate response to DNA damage
Watanya Trakarnphornsombat and Hiroshi Kimura
DOI: 10.1242/jcs.260698

Editor: Maria Carmo-Fonseca

Review timeline

Original submission:	9 October 2022
Editorial decision:	16 November 2022
First revision received:	21 March 2023
Accepted:	23 March 2023

Original submission

First decision letter

MS ID#: JOCES/2022/260698

MS TITLE: Live-Cell Tracking of γ-H2AX Kinetics Reveals the Distinct Modes of ATM and DNA-PK in Immediate Response to DNA Damage

AUTHORS: Watanya Trakarnphornsombat and Hiroshi Kimura
ARTICLE TYPE: Research Article

We have now reached a decision on the above manuscript.

To see the reviewers' reports and a copy of this decision letter, please go to: https://submit-jcs.biologists.org and click on the 'Manuscripts with Decisions' queue in the Author Area. (Corresponding author only has access to reviews.)

As you will see, the reviewers raise a number of substantial criticisms that prevent me from accepting the paper at this stage. They suggest, however, that a revised version might prove acceptable, if you can address their concerns. If you think that you can deal satisfactorily with the criticisms on revision, I would be pleased to see a revised manuscript. We would then return it to the reviewers.

Please ensure that you clearly highlight all changes made in the revised manuscript. Please avoid using 'Tracked changes' in Word files as these are lost in PDF conversion.

I should be grateful if you would also provide a point-by-point response detailing how you have dealt with the points raised by the reviewers in the 'Response to Reviewers' box. Please attend to all of the reviewers' comments. If you do not agree with any of their criticisms or suggestions please explain clearly why this is so.

Reviewer 1

Advance summary and potential significance to field

In this manuscript, Trakarnphornsombat and Kimura present live-cell microscopy data of γH2AX dynamics at 405nm laser-induced DSBs. Using fluorescently labelled antigen-binding fragments, they analyse the kinetic behaviour of γH2AX in different cellular contexts to explore the impact of ATM, DNA-PK and MOF on γH2AX immediately after induction of DSBs. The work is mostly descriptive and lack clear mechanistic or conceptual advance over the current state-of-the-art.

© 2023. Published by The Company of Biologists under the terms of the Creative Commons Attribution License (https://creativecommons.org/licenses/by/4.0/).

Actually, the main conclusions of the manuscript are either confirmation of previously known processes or negative results (more obvious in the experiments involving depletion of MOF). Nevertheless, this is a sensibly executed piece of work that describes dynamic parameters of the DNA damage response that may be of interest to a specialized audience. To strengthen the study, a set of additional data and necessary controls should be included, as detailed below.

Comments for the author

Specific comments:

1. Appropriate statistics analyses to assess the significance of the differences obtained must be included in all datasets. Conclusions must be revised according to the statistical significance of the corresponding data.

2. Page 5 – lines 159-161. This should be experimentally tested using the ATR inhibitor that the authors used in other experiments.

3. Page 6 – lines 195-197. To support the involvement of ATR and further strengthen the conclusions, the impact of MOF KD on the cell cycle progression should be analysed.

4. The γH2AX curves in Figs 2C (W/ NU7441) and 3H (control W/ NU7441) should have shown similar behaviour as both refer to a similar condition. However, they are considerably different from each other. The reasons for the difference should be clarified and the conclusions revised.

5. Fig. 5 – Positive controls showing that the pre-extraction successfully resulted in the loss of chromatin-unbound proteins (namely ATM and DNA-PK) must be included in the manuscript.

<u>Reviewer 2</u>

Advance summary and potential significance to field

DNA double strand breaks (DSBs) are the most serious form of DNA damage inducing genetic alterations and/or cell death. Phosphorylation of histone H2AX, or gamma-H2AX, is one of the earliest responses of cells against DSBs. Gamma-H2AX forms nuclear foci to serve as a scaffold for the accumulation of various DNA repair factors at damaged sites. PI3 kinase family, including ATM, ATR and DNA-PK has been shown to be involved in the phosphorylation of H2AX. However, how these kinases are regulated to phosphorylate H2AX just after the induction of DSBs are remained to be clarified.

In this study, the authors showed the similar dynamics of ATM and the phosphorylation of H2AX after induction of DSBs by laser microbeam using gamma-H2AX Fab they developed. Then, they examined the effect of DNA-PK, ATM and ATR inhibitors on gamma-H2AX Fab dynamics in ATM deficient and proficient cells to show that DNA-PK activity is critical in the phosphorylation of H2AX. The authors investigated the involvement of MOF, a histone acetyltransferase phosphorylated by ATM, in the phosphorylation of H2AX after induction of DNA damage, and found the role of MOF as a regulator of ATM, not for the phosphorylation of H2AX. Then, the authors examined the mobility of ATM and Ku-80 upon DNA damage, and showed that ATM binds to and dissociates form chromatin, and more stably binds to chromatin after induction of DSBs.

From these findings, they suggest distinct actions of ATM and DNA-PK that plays a primary role in immediate early gamma-H2AX accumulation.

Comments for the author

This is an interesting manuscript paper studying the role of ATM, DNA-PK in early γ-H2AX kinetics using live-cell tracking technique. The manuscript is well written, and the results presented in this study are very compelling and may provide important new insights into the molecular mechanisms of early DNA damage response. There are several points clarified before publication.
1) Fig. 1: To confirm the γ-H2AX kinetics obtained with 11-4 and BIVA cells, the author should examine that of 11-4 cells treated with an ATM kinase inhibitor.

© 2023. Published by The Company of Biologists under the terms of the Creative Commons Attribution License (https://creativecommons.org/licenses/by/4.0/).

2) Page 4, line 147 and 148: “When DNA-PK activity is inhibited, ATM can phosphorylate H2AX in 60 min.”: Where are the data? Or is this from the reference?

3) Fig. 3: Figs. 3C and D are same. Are the data obtained by immunoblotting and immunofluorescence same? Representative images of immunofluorescence analysis can be shown. The fluorescence staining of H4K16ac should be described in Methods.

4) Page 6, line 226: “a permeabilized cell system”: The authors suggest the possibility that a chromatin bound fraction of ATM remains but freely diffusing DNA-PK is mostly extracted during permeabilization. The authors can show this by examining the localization of ATM and DNA-PK by GFP fusion proteins or immunofluorescence staining.

5) Page 7, line 241: “Fig. 5D”. should be “Fig. 5D-F”.

6) Fig. S6A (upper panel): In the legend, they wrote that they used AT5BIVA cells expressing EGFP-ATM but at line 247, page 7 in the text they wrote that 11-4 cells were used.

First revision

Author response to reviewers' comments

A point-by-point response to reviewers’ comments

In the revised version, we revised all graphs by increasing the number of cells and replicates and added statistical significance. We also added the following figures.

Fig. 2D,E. γ-H2AX in AT5BIVA cells treated with AZ20 and/or NU7441
Fig. 2F. γ-H2AX in AT5BIVA cells treated with AZ20 and/or NU7441
Fig. 2G. ATM dynamics in KU55933 or NU7441
Fig. S1B,C. γ-H2AX dynamics in 3 independent experiments
Fig. S4A. Images for H4K16ac dynamics
Fig. S5D. Number of apoptotic cells in MOF knockdown cells
Fig. S5E,F. Cell cycle analysis of the MOF knockdown cells
Fig. S6. The localization of ATM and Ku80 in permeabilized cells

The reviewers’ comments are indicated in blue and the changed parts in the text are expressed in red.

Reviewer #1

General comments:

In this manuscript, Trakarnphornsombat and Kimura present live-cell microscopy data of γH2AX dynamics at 405nm laser-induced DSBs. Using fluorescently labelled antigen-binding fragments, they analyse the kinetic behavior of γH2AX indifferent cellular contexts to explore the impact of ATM, DNA-PK and MOF on γH2AX immediately after induction of DSBs. The work is mostly descriptive and lack clear mechanistic or conceptual advance over the current state-of-the-art. Actually, the main conclusions of the manuscript are either confirmation of previously known processes or negative results (more obvious in the experiments involving depletion of MOF). Nevertheless, this is sensibly executed piece of work that describes dynamic parameters of the DNA damage response that may be of interest to a specialized audience. To strengthen the study, a set of additional data and necessary controls should be included, as detailed below.

We appreciate your positive and constructive comments.

Major points:

1. Appropriate statistics analyses to assess the significance of the differences obtained must be

© 2023. Published by The Company of Biologists under the terms of the Creative Commons Attribution License (https://creativecommons.org/licenses/by/4.0/).

included in all datasets. Conclusions must be revised according to the statistical significance of the corresponding data.

2. The γH2AX curves in Figs 2X (W/ NU7441) and 3H (control W/ NU7441) should have shown similar behavior as both refer to a similar condition. However, they are considerably different from each other. The reasons for the difference should be clarified and the conclusions revised.

Thank you for the comments. We now indicated the statistical significance in the figures and provided the actual *p* values for accumulation kinetics in the figure legends. Also, we now combined all the data from 2-5 replicates to obtain averages with reduced noise, whereas we used the data from one representative replicate in the original version. The numbers of total cells and replicates are indicated in the figures and legends. The variations in independent experiments are also shown in Fig. S1B,C. After increasing the number of cells and statistical analysis, a subtle difference was observed in γ-H2AX accumulation kinetics between 11-4 and AT5BIVA cells (Fig. 1C).

By increasing the number of cells, the curves become smoother and internally more consistent (e.g., Fig. 2B,C for untreated and Fig. 3E–H for control shRNA expression). As a result, we no longer found the effects on γ-H2AX accumulation kinetics by MOF knockdown (Fig. 3E-H) (see the response to point 3 below for the detail).

Page 2, lines 33-38.

"Ku80, a DNA-PK subunit, diffused freely in the nucleus without DNA damage, whereas ATM repeatedly bound to and dissociated from chromatin. The accumulation of ATM at damage sites were regulated by a histone H4K16 acetyltransferase, but its accumulation was not necessarily reflected in γ-H2AX level. These results suggest distinct actions of ATM and DNA-PK that plays a primary role in immediate γ-H2AX accumulation."

Page 3, lines 108-109.

"The relative fluorescence intensities of γ-H2AX Fab in the irradiated area were measured and plotted from three independent experiments (Fig.1C and S1B,C)."

Page 6, lines 208-217.

"In both 11-4 and AT5BIVA cells, either the absence or presence of the DNA-PK inhibitor, NU7441, γ-H2AX Fab kinetics were similar in the MOF knockdown and the scrambled shRNA control cells (Fig. 3E-H), suggesting that MOF did not affect the immediate γ-H2AX formation through ATM and DNA-PK in response to DNA damage. However, the accumulation of EGFP- ATM in the irradiated area was significantly reduced in MOF-knockdown cells than in the scrambled control without and with NU7441 (Figs. 3I,J). These results imply that MOF facilitates ATM accumulation at damage sites, in agreement with MOF being a regulator of ATM (Gupta et al., 2005); however, the reduced amount of accumulated ATM by MOF knockdown could still mediate γ-H2AX formation in the early response."

Page 8, lines 288-295

"In both ATM-proficient and -deficient cells, γ-H2AX accumulated immediately after irradiation and reached a broad peak at ~100–200 s before gradually decreasing, which may be associated with progression of DNA repair (Bouquet et al., 2006; MacPhail et al., 2003; Mah et al., 2010). Although γ-H2AX accumulation appear to be slightly higher in ATM-proficient cells than ATM-deficient cells, ATM does not appear to have a major role in the immediate γH2AX formation upon DNA damage, in contrast to the later responses in which ATM has a critical role (Caron et al., 2015; Kuhne et al., 2004; Lobrich and Jeggo, 2005; Loucas and Cornforth, 2004; Riballo et al., 2004; Stiff et al., 2004)."

Page 13, lines 507-513

"For the dynamics of γ-H2AX accumulation, Student's *t*-test (two-tailed) or One-way ANOVA test with Tukey test as the post hoc analysis was performed at 105, 210, and 315 sec. For FRAP experiments, the Student's *t*-test (two-tailed) was performed at 0.5, 1, 2, and 4 sec. For immunofluorescence

© 2023. Published by The Company of Biologists under the terms of the Creative Commons Attribution License (https://creativecommons.org/licenses/by/4.0/).

data, One-way ANOVA test with Tukey test as the post hoc analysis was performed. IBM SPSS Statistics for Windows, version 22 (IBM Corp.) was used for statistical analysis. Statistical significance was indicated by asterisks (*, $p < 0.05$; **, $p < 0.01$; ***, $p < 0.001$)."

2. Page 5 – lines 159-161. This should be experimentally tested using the ATR inhibitor that the authors used in other experiments.

Thank you for the comment. In the revised version, the γ-H2AX accumulation in AT5BIVA cells treated with ATR and DNA-PK inhibitors was added (Fig. 2D–E).

Page 5, lines 163–166.

"When AT5BIVA cells were treated with AZ20 and NU7441 to inhibit both ATR and DNA-PK, γ-H2AX level was reduced to the same level as control cells without DNA damage (Figs. 2D- E), which suggests that the low level of γ-H2AX accumulation in NU7441-treated AT5BIVA cells (Figs. 2A,C) was mediated by ATR (Boe et al., 2018)."

3. Page 6 – lines 195-197. To support the involvement of ATR and further strengthen the conclusions, the impact of MOF KD on the cell cycle progression should be analysed.

Thank you for the comment. After increasing the number of cells by combining all replicates and additional experiments, the effect of MOF knockdown on γ-H2AX accumulation kinetics was no longer observed. Interestingly, however, EGFP-ATM accumulation was affected by MOF knockdown (Fig. 3I,J). We, therefore, changed the description and interpretation of the MOF knockdown data according to the new results. Together with the increase of MOF accumulation induced by DNA-PK inhibitor (Fig. 2G), we were able to make an interesting discussion. We really appreciate for the reviewer's comment to make a more robust analysis and not to make a wrong conclusion. We also added data for the impact of MOF KD on cell cycle progression and apoptosis.

Page 6, lines 203-217.

"Given that MOF knockdown increased apoptotic cells from 1.5% to 18.7% in 11-4 cells and 0.5% to 9.3% in AT5BIVA cells (Fig. S5D) (Li et al., 2012; Thomas et al., 2008), we analyzed cells that showed normal nuclear shape regardless of the cell-cycle phase for a laser irradiation assay. MOF knockdown subtly affected the cell cycle progression, increasing G2 and G1 fractions in 11-4 and AT5BIVA cells, respectively, but the majority of cells (47-63%) were in S phase in all cell types (Fig. S5E,F). In both 11-4 and AT5BIVA cells, either the absence or presence of the DNA-PK inhibitor, NU7441, γ-H2AX Fab kinetics were similar in the MOF knockdown and the scrambled shRNA control cells (Fig. 3E–H), suggesting that MOF did not affect the immediate γ-H2AX formation through ATM and DNA-PK in response to DNA damage. However, the accumulation of EGFP-ATM in the irradiated area was significantly reduced in MOF-knockdown cells than in the scrambled control without and with NU7441 (Figs. 3I,J). These results imply that MOF facilitates ATM accumulation at damage sites, in agreement with MOF being a regulator of ATM (Gupta et al., 2005); however, the reduced amount of accumulated ATM by MOF knockdown could still mediate γ-H2AX formation in the early response."

4. Fig. 5 – Positive controls showing that the pre-extraction successfully resulted in the loss of chromatin-unbound proteins (namely ATM and DNA-PK) must be included in the manuscript

Thank you for the comment. We now included the data showing that the permeabilization assay resulted in the loss of DNA-PK and the retention of ATM in the nucleus by using the immunofluorescence staining and the EGFP-fusion protein (Fig. S6A,B).

Page 7, lines 251-260.

"Immunostaining and EGFP fluorescence indeed confirmed that in permeabilized cells a fraction of ATM remains in the nucleus while Ku80 is largely extracted (Fig. S6A,B). Thus, in permeabilized cells, only ATM-proficient cells contain H2AX phosphorylation activity in response to laser-induced DNA damage (Fig. 5A). Under MOF knockdown, the accumulation of γ-H2AX Fab in 11-4 cells was significantly impaired particularly at the later time points (Fig. 5D,E). This differs from the

© 2023. Published by The Company of Biologists under the terms of the Creative Commons Attribution License (https://creativecommons.org/licenses/by/4.0/).

observation in living cells in which MOF knockdown results in little or no effect on γ-H2AX accumulation kinetics (Fig. 3E-H). As proteins dissociated from chromatin can diffuse out from the nucleus in permeabilized cells, the effect of reduced ATM binding rate by MOF knockdown may become apparent in permeabilized cells."

Reviewer #2

General comments:

This is an interesting manuscript paper studying the role of ATM, DNA-PK in early γ-H2AX kinetics using live-cell tracking technique. The manuscript is well written, and the results presented in this study are very compelling and may provide important new insights into the molecular mechanisms of early DNA damage response. There are several points clarified before publication.

We appreciate your positive and constructive comments.

Major points:

1. To confirm the γ-H2AX kinetics obtained with 11-4 and BIVA cells, the author should examine that of 11-4 cells treated with an ATM kinase inhibitor.

Thank you for the comment. The result with the ATM inhibitor, KU55933, was not what we expected. γ-H2AX kinetics of 11-4 cells treated with an ATM kinase inhibitor differed from that of ATM-deficient AT5BIVA cells (Fig. 2F). γ-H2AX exhibited more rapid accumulation in KU55933 and EGFP-ATM accumulation was unaffected by KU55933 (Fig. 2G). The results suggested that the ATM inhibitor does not phenocopy the ATM-deficient cells. With this and new data, we now discuss the relationship between γ-H2AX accumulation and ATM accumulation.

Page 5, lines 167-180.

"To confirm that the early γ-H2AX dynamics were similar regardless of the presence of ATM, 11-4 cells were treated with the ATM inhibitor, KU55933, before and during laser-irradiation. Unexpectedly, γ-H2AX accumulated more rapidly in KU55933-treated 11-4 cells compared to that of untreated 11-4 and AT5BIVA cells (Fig. 2F), whereas EGFP-ATM accumulated similarly without and with KU55933 (Fig. 2G, blue and green curves). This indicates that using the ATM-specific inhibitor does not phenocopy the ATM protein-deficient cells, as previously suggested (Choi et al., 2010). The mechanism of how the chemical inhibition of ATM stimulates γ-H2AX formation in early response remains unknown, but DNA-PK accumulate slightly more in KU55933 (see below). In the presence of the DNA-PK inhibitor, NU7441, EGFP-ATM accumulated much more (Fig. 2G), suggesting that the inhibition of one of ATM and DNA-PK can facilitate the accumulation of another by a compensation mechanism. However, in the presence of the DNA-PK inhibitor, a substantial time-lag was observed for γ-H2AX accumulation kinetics despite more ATM was accumulated. From this observation, it is likely that the accumulation and activation of ATM are not coupled."

2. Page 6, line 226: "a permeabilized cell system": The authors suggest the possibility that a chromatin bound fraction of ATM remains but freely diffusing DNA-PK is mostly extracted during permeabilization. The authors can show this by examining the localization of ATM and DNA-PK by GFP fusion proteins or immunofluorescence staining.

Thank you for the comment. We now included the data showing that the permeabilization assay resulted in the loss of DNA-PK and the retention of ATM in the nucleus by using the immunofluorescence staining (Fig. S6A) and the EGFP-fusion protein (Fig. S6B).

Page 7, lines 251-260.
"Immunostaining and EGFP fluorescence indeed confirmed that in permeabilized cells a fraction of ATM remains in the nucleus while Ku80 is largely extracted (Fig. S6A,B). Thus, in permeabilized cells, only ATM-proficient cells contain H2AX phosphorylation activity in response to laser-induced DNA damage (Fig. 5A). Under MOF knockdown, the accumulation of γ-H2AX Fab in 11-4 cells was significantly impaired particularly at the later time points (Fig. 5D,E). This differs from the

© 2023. Published by The Company of Biologists under the terms of the Creative Commons Attribution License (https://creativecommons.org/licenses/by/4.0/).

observation in living cells in which MOF knockdown results in little or no effect on γ-H2AX accumulation kinetics (Fig. 3E-H). As proteins dissociated from chromatin can diffuse out from the nucleus in permeabilized cells, the effect of reduced ATM binding rate by MOF knockdown may become apparent in permeabilized cells."

Minor points:

1. Page 4, line 147 and 148: "When DNA-PK activity is inhibited, ATM can phosphorylate H2AX in 60 min.": Where are the data? Or is this from the reference?

We apologize for the confusion. We now referred to Fig. S3.

Page 4, lines 151-152.

"When DNA-PK activity is inhibited, ATM can phosphorylate H2AX in 60 min (Fig. S3)."

2. Fig. 3: Figs. 3C and D are same. Are the data obtained by immunoblotting and immunofluorescence same? Representative images of immunofluorescence analysis can be shown. The fluorescence staining of H4K16ac should be described in Methods.

First, we apologize to use the incorrect graph for Fig. 3D. We corrected. The data obtained from immunoblotting and immunofluorescence showed similar results that H4K16ac level decreased in MOF knockdown cells. The representative images of the immunofluorescence analysis are shown in Fig. S5A-C.

Page 6, lines 198-203.

"Immunoblotting showed that MOF-specific shRNA expression lowered MOF and H4K16ac levels to 10%-20% and 20%-40%, respectively, relative to the expression of scramble shRNA control (Figs. 3A-C). Immunofluorescence confirmed the decrease in H4K16ac by MOF- specific shRNA expression (Fig. 3D and Fig. S5A-C) and showed that γ-H2AX was still formed by ETP treatment for 20 min in MOF knockdown cells (Fig. S5A-C)"

3. Page 7, line 241: "Fig. 5D". should be "Fig. 5D-F".

Thank you for the comment. We corrected.

Page 7, lines 254-260.

"Under MOF knockdown, the accumulation of γ-H2AX Fab was significantly impaired particularly at the later time points in 11-4 cells but was unchanged in AT5BIVA (Fig. 5D-F), suggesting the function of MOF in ATM. This data differs from the observation in living cells in which MOF knockdown results in little or no effect on γ-H2AX accumulation kinetics (Fig. 3E-H). As proteins dissociated from chromatin can diffuse out from the nucleus in permeabilized cells, the effect of reduced ATM binding rate by MOF knockdown may become apparent in permeabilized cells."

4. Fig. S6A (upper panel): In the legend, they wrote that they used AT5BIVA cells expressing EGFP-ATM, but at line 247, page 7 in the text they wrote that 11-4 cells were used.

We apologize for our carelessness. We corrected.

Page 7, lines 265-267.

"Within several seconds, γ-H2AX was accumulated in irradiated areas in sensitized AT5BIVA cells expressing EGFP-ATM and was then soon enriched in unirradiated areas in the nucleus (Fig. S7A)."

© 2023. Published by The Company of Biologists under the terms of the Creative Commons Attribution License (https://creativecommons.org/licenses/by/4.0/).

Second decision letter

MS ID#: JOCES/2022/260698

MS TITLE: Live-Cell Tracking of γ-H2AX Kinetics Reveals the Distinct Modes of ATM and DNA-PK in Immediate Response to DNA Damage

AUTHORS: Watanya Trakarnphornsombat and Hiroshi Kimura

ARTICLE TYPE: Research Article

I am happy to tell you that your manuscript has been accepted for publication in Journal of Cell Science, pending standard ethics checks.

© 2023. Published by The Company of Biologists under the terms of the Creative Commons Attribution License (https://creativecommons.org/licenses/by/4.0/).
